# Bioinformatics approach for discovery of potential lead compound of NSP6 of SARS-CoV-2 using structure based virtual screening and molecular dynamics simulations

**DOI:** 10.1038/s41598-025-22409-0

**Published:** 2025-10-27

**Authors:** Mohammed M. Salama, Medhat W. Shafaa, Mohamed E. El-Nagdy, Mohamed E. Hasan

**Affiliations:** 1 Physics Department, Medical Biophysics Division, Faculty of Science, Helwan University, Cairo, Egypt; 2 Bioinformatics Department, Genetic Engineering and Biotechnology Research Institute, University of Sadat City, Sadat City, 32897 Egypt

**Keywords:** MMPBSA, SARS-CoV-2, Docking, Molecular dynamics simulation, NSP6, ZINC20, Biotechnology, Biophysics, Computational biophysics

## Abstract

Non-Structural Protein 6 (NSP6) is a crucial protein for SARS-CoV-2 as it performs a vital role in the replication and transcription of the virus. NSP6 plays a role in the stress response of the endoplasmic reticulum through binding to the sigma receptor 1 (SR1). Therefore, NSP6 is an interesting target for fighting SARS-CoV-2. The best model of the tertiary structure of NSP6 was predicted using the AlphaFold server, then this model was refined using the DeepRefiner server to construct a good-quality model. The current study utilized the virtual screening of the ZINC20 database for the identification of possible inhibitors using the computational docking Autodock Vina program and the post-docking analysis of energy calculations and interactions followed by ADMET studies which were widely used in potential hit identification and lead optimization. From the final hits, our study revealed the best eight compounds that have more potential to be considered as lead compounds for inhibition of a vital role of NSP6 in the replication and transcription of the virus with docking score and binding energy (E_score) with values ranging from − 49.64 to − 43.13 and all these compounds displayed no cellular cytotoxicity. These complexes in addition to the apoprotein were validated and analyzed using molecular dynamic simulations (MDS) for 100 ns. The NSP6-ZINC-141,457,420, NSP6-ZINC-075486396 complexes, revealed the highest complexes in H-bonds formation, NSP6-ZINC-018529632 complex was found to have the lowest binding energy score and NSP6-ZINC-089639800, NSP6-ZINC-157,610,683, NSP6-ZINC-075484852, NSP6-ZINC-016611776, and NSP6-ZINC-141,457,420 complexes presented no a significant deviation from the NSP6-apo protein in the Rg and SASA analysis and revealed promising performance in the RMSD and RMSF. This study exhibited that ZINC-141,457,420, ZINC-075486396, and ZINC-018529632 ligands have a promising potential for development as potential vaccines inhibiting SARS-CoV-2 NSP6 protein.

## Introduction

Severe acute respiratory syndrome coronavirus 2 (SARS-CoV-2) is a novel virus that caused a pandemic that infected hundreds of millions of patients and caused the death of millions all over the globe^1,2^. The genome of SARS-CoV-2 comprises 26 to 32 kb containing 6–11 open reading frames (ORFs) that encode 9680 amino acid polyproteins^3^. ORF1 includes most of the whole genome of SARS-CoV-2 with 67% and encodes 16 nonstructural proteins (NSPs), that are milestones in the replication and transcription of the virus. The leftover ORFs encode the accessory and structural proteins. In addition, the genome of SARS-CoV-2 includes four essential structural proteins the envelope (E), spike (S), nucleocapsid (N) and membrane (M) proteins^4,5^.

NSP6 is one of the most attractive SARS-CoV-2 NSPs for drug targeting, though it does not have a verified crystal structure till now which allows the researchers to use bioinformatical tools to predict its tertiary structure model for further studies^6,7^. NSP6 is composed of 290 residues, it is found to play important roles in the cycle of infection, protection, and replication processes of the SARS-CoV-2^8–10^. In addition, NSP6 is found to have a crucial role in the replication-transcription complexes (RTCs) assembly with NSP3 and NSP4 by increasing the double-membrane vesicles (DMVs) production^11,12^. Also, it is found that NSP6 is effective on the endoplasmic reticulum (ER) by its interactions with the sigma receptor of the ER^7,13,14^.

Structural bioinformatics plays a crucial role in biomedical research, particularly in drug and vaccine development. By predicting the three-dimensional structures of biomolecules, computational approaches offer a viable alternative to traditional experimental techniques such as NMR spectroscopy and X-ray crystallography. These methods not only reduce the high cost, time, and effort associated with laboratory-based validation but also accelerate the discovery of potential therapeutic targets. Due to the lack of an effective and suitable drugs for SARS-CoV-2, bioinformatics tools could help in finding a successful drug in low periods and cost^15,16^. Some researchers made studies to understand NSP6 protein, and to predict the most suitable vaccine fighting SARS-CoV-2 using bioinformatics^6–10^. Molecular dynamic simulation (MDS) is a process that aims to analyze the dynamics of macromolecules in a simulated well-controlled biophysical environment, through this computational process the physical interactions in the system through the observed flexibility of the structures along the simulation time. Also, the MDS process aims to study the interactions between the ligand and the active sites of the NSP6 protein in addition to estimating the stability of these interactions^7^.

In this study, we aimed to utilize multi-techniques like protein structure prediction, epitope prediction and vaccine design, molecular docking, and ADMET prediction. The main emphasis is the utilization of advanced tools and techniques on the target protein to develop next-generation drugs and vaccines to combat this disease.

## Results

### Structure modelling of NSP6

**Fig. 1:**
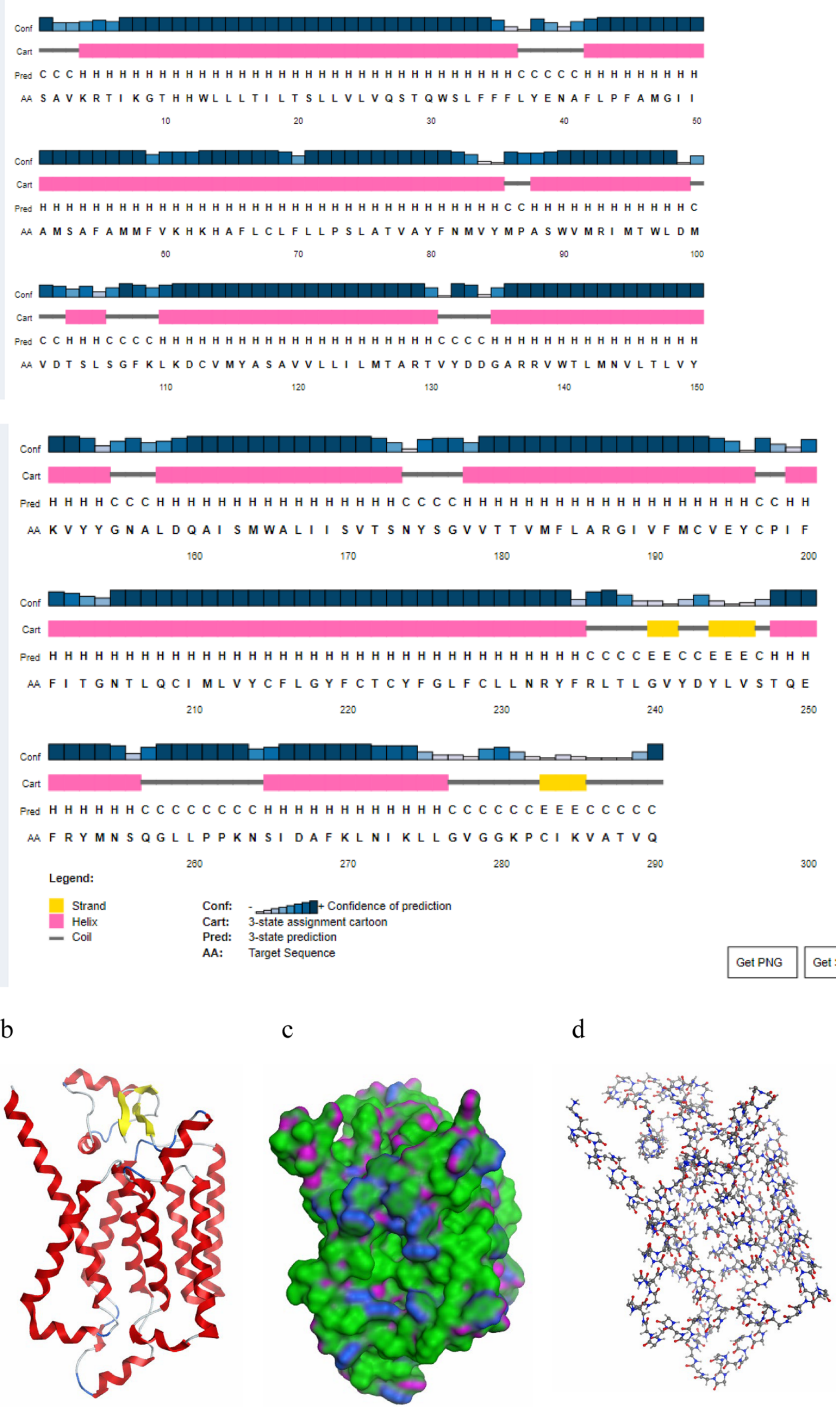
(**a**) The predicted secondary structure of NSP6. The best predicted three-dimensional structure of NSP6 protein of SARS-CoV-2 using AlphaFold server which is generated by Autodock Vina. (**b**) The ribbon view of the best-predicted model, (**c**) the solvent-accessible surface view, showing the exposed regions, and (**d**) The atomic representation of the protein.

The secondary structure of the NSP6 protein is predicted using PSIPRED server, the predicted secondary structure from the PSIPRED server may be composed of 77.93% α-helices, 2.76% β-strands, and 19.31% coils. then the tertiary structure model of SARS-CoV-2 NSP6 protein is predicted using the Alphafold server. In addition, the model is refined using the DeepRefiner server to get an improved model with high quality. The predicted model shows that NSP6 structure is composed of two antiparallel beta sheets, sixteen turns, one C-terminal and fourteen alpha helices which include 8 transmembrane helices. The SAVES server was used to estimate the overall quality of the predicted model according to the ERRAT server is 99.64%, and according to the PROCHECK server, the Ramchandran plot revealed that the protein has 94.4% in the core regions and 5.6% in the allowed region while no residues are found in the disallowed region (Fig. 1).

### Binding site prediction and virtual screening

#### Prediction of cytotoxic T lymphocytes epitope

Using NetCTL 1.2 server, 22 CTL epitopes were predicted that have binding affinities to MHC-I. In addition, studying the non-toxicity, non-allergenicity, and antigenicity properties of the epitopes, only 3 epitopes were predicted (Table 1).

**Table 1:** List of the filtered CTL epitopes using NetCTL 1.2 server.

	Epitope	Antigenicity	Allergenicity	Nontoxicity	Immunogenicity
1	QSTQWSLFF	0.9087	NON-ALLERGEN	Non-Toxin	0.01505
2	STQWSLFFF	0.7828	NON-ALLERGEN	Non-Toxin	0.18095
3	ILMTARTVY	1.0970	NON-ALLERGEN	Non-Toxin	0.12576

#### Prediction of Helper T lymphocytes epitope

Using IEDB MHC-II server, a total of 25 HTL epitopes were predicted for mouse MHC class II alleles H2-IAb, H2-IAd and H2-IEd alleles according to the antigenicity, non-toxicity, binding affinity and non-allergenicity (Table 2).

**Table 2:** List of the filtered HTL epitopes using IEDB MHC-II server.

	Epitope	Antigenicty	Allergenity	Nontoxicity	Immunogenicity
1	CLLNRYFRLTLGVYD	0.7081	NON-ALLERGEN	Non-Toxin	0.28187
2	NAFLPFAMGIIAMSA	0.9681	NON-ALLERGEN	Non-Toxin	0.08131
3	LPFAMGIIAMSAFAM	1.0879	NON-ALLERGEN	Non-Toxin	0.06306
4	PASWVMRIMTWLDMV	0.5073	NON-ALLERGEN	Non-Toxin	0.20657
5	FGLFCLLNRYFRLTL	1.1133	NON-ALLERGEN	Non-Toxin	0.25664
6	ENAFLPFAMGIIAMS	1.1389	NON-ALLERGEN	Non-Toxin	0.3083
7	YENAFLPFAMGIIAM	1.1062	NON-ALLERGEN	Non-Toxin	0.40423
8	FFLYENAFLPFAMGI	0.8191	NON-ALLERGEN	Non-Toxin	0.23681
9	LYENAFLPFAMGIIA	0.807	NON-ALLERGEN	Non-Toxin	0.35957
10	LFCLLNRYFRLTLGV	0.903	NON-ALLERGEN	Non-Toxin	0.20162
11	MPASWVMRIMTWLDM	0.5001	NON-ALLERGEN	Non-Toxin	0.19004
12	LFFFLYENAFLPFAM	0.6202	NON-ALLERGEN	Non-Toxin	0.48512
13	FFFLYENAFLPFAMG	0.8851	NON-ALLERGEN	Non-Toxin	0.28556
14	FLYENAFLPFAMGII	0.8142	NON-ALLERGEN	Non-Toxin	0.31691
15	VVLLILMTARTVYDD	0.6304	NON-ALLERGEN	Non-Toxin	0.14584
16	FLPFAMGIIAMSAFA	1.0725	NON-ALLERGEN	Non-Toxin	0.08629
17	AFLPFAMGIIAMSAF	1.0499	NON-ALLERGEN	Non-Toxin	0.01458
18	ISMWALIISVTSNYS	0.5978	NON-ALLERGEN	Non-Toxin	0.20594
19	AISMWALIISVTSNY	0.5847	NON-ALLERGEN	Non-Toxin	0.04404
20	LFLLPSLATVAYFNM	0.6553	NON-ALLERGEN	Non-Toxin	0.04828
21	CLFLLPSLATVAYFN	0.6117	NON-ALLERGEN	Non-Toxin	0.05155
22	FLCLFLLPSLATVAY	0.6654	NON-ALLERGEN	Non-Toxin	0.01338
23	LCLFLLPSLATVAYF	0.5024	NON-ALLERGEN	Non-Toxin	0.05552
24	DQAISMWALIISVTS	0.536	NON-ALLERGEN	Non-Toxin	0.21851
25	LPFAMGIIAMSAFAM	1.0879	NON-ALLERGEN	Non-Toxin	0.06306

#### Prediction of linear B‑cell epitopes

B-cell epitopes were predicted using the ABCpred server. Using binding score cut-off > 0.8 for the 14-mer predicted B-cell epitopes alongside being non-allergenic, high antigenic and non-toxic, only 5 B-cell epitopes were predicted (Table 3).

**Table 3:** List of the filtered B-cell epitopes using ABCpred server.

Serial no.	Sequence	Start position	Binding score
1	CFLGYFCTCYFGLF	215	0.87
2	KLKDCVMYASAVVL	109	0.84
3	FGLFCLLNRYFRLT	225	0.81
4	VLTLVYKVYYGNAL	145	0.81
5	RVWTLMNVLTLVYK	138	0.81

### Virtual screening and molecular docking

**Fig. 2:**
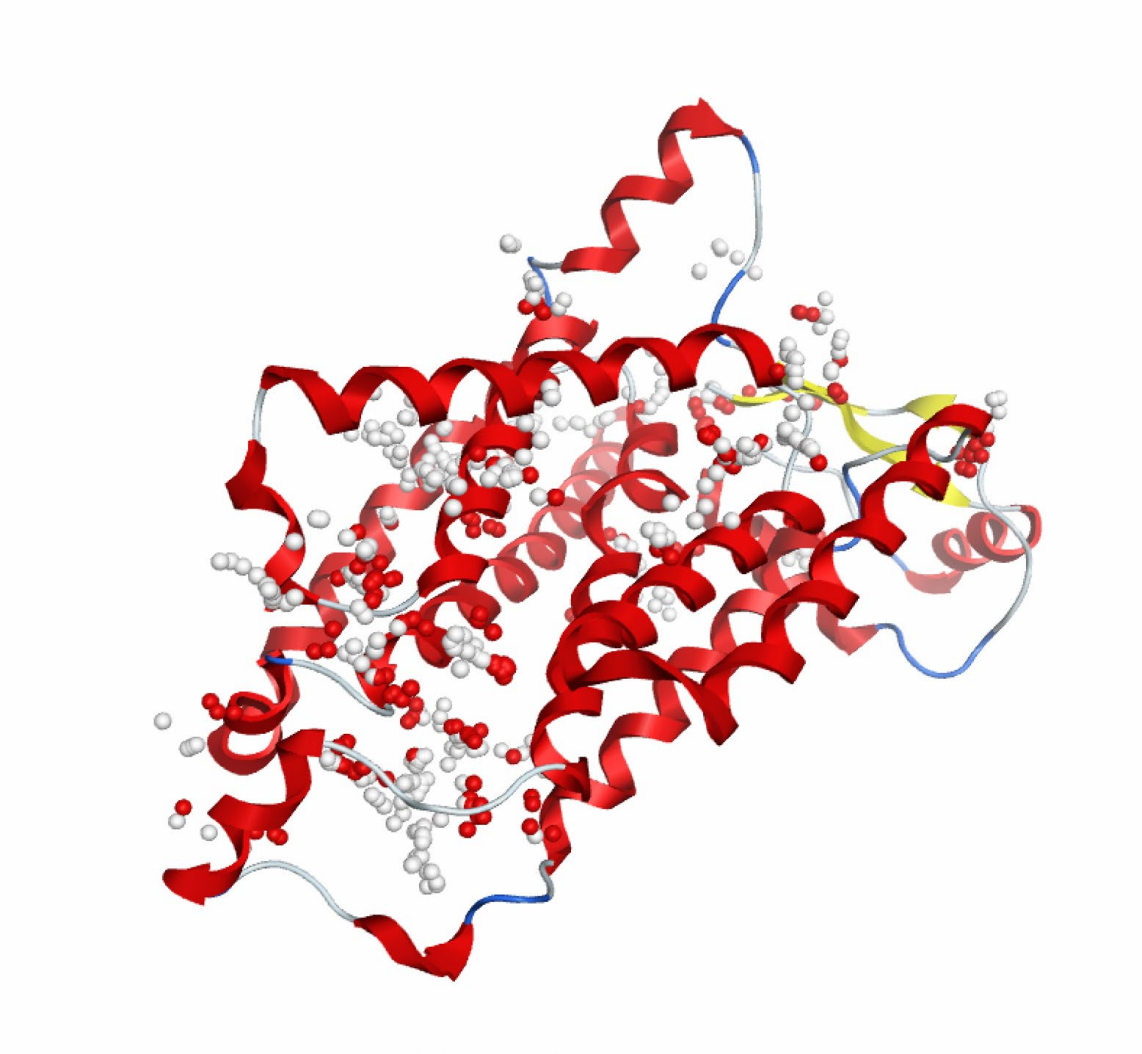
Three-dimensional (3D) protein structures, showing the binding sites of SARS-CoV-2 NSP6 predicted by Autodock Vina.

**Fig. 3:**
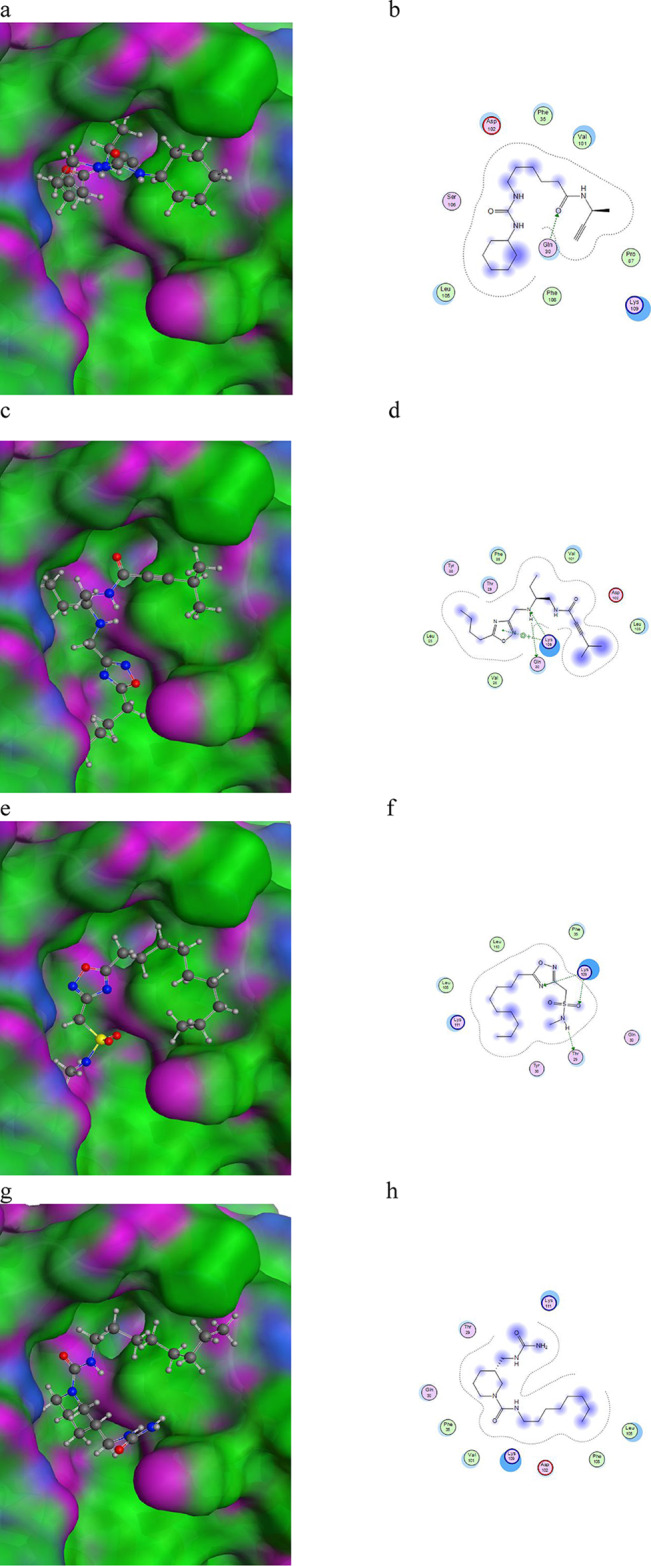

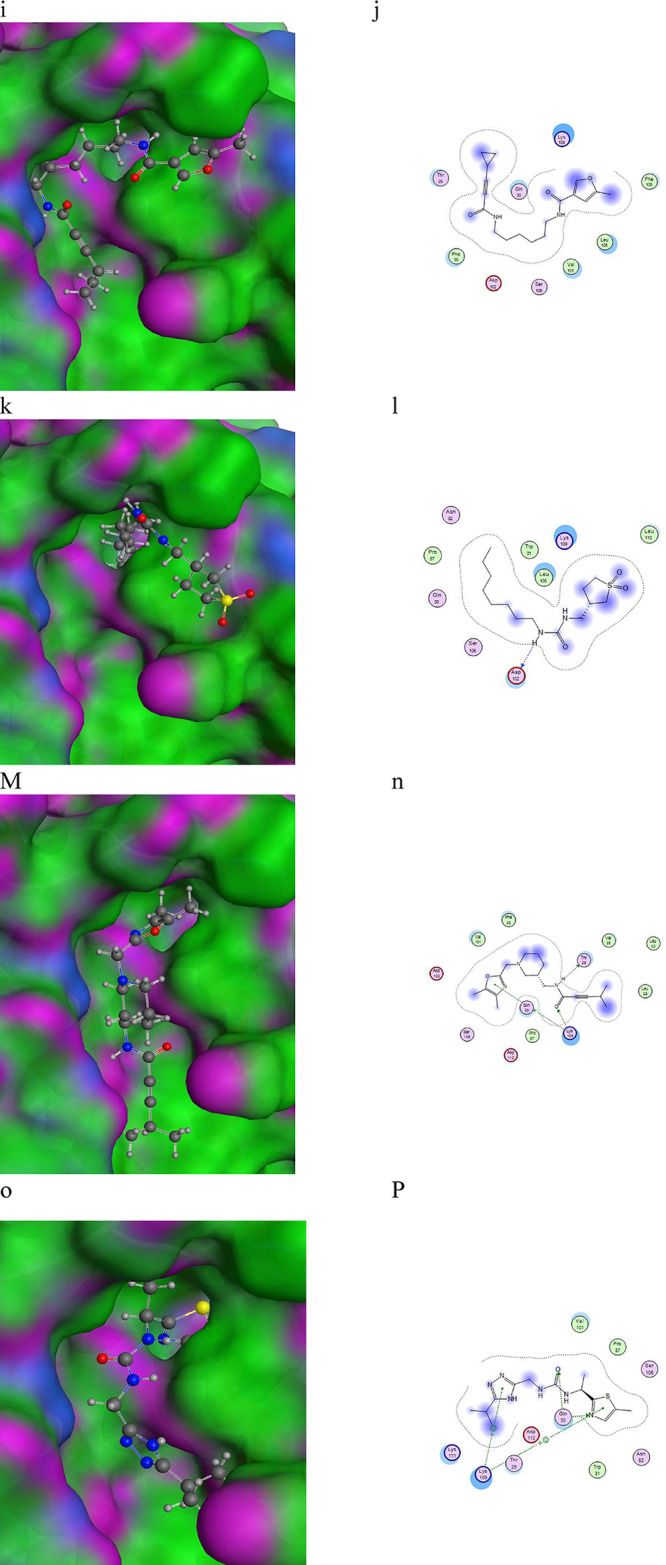
Docking interactions of (**a**, **b**) NSP6-ZINC-018529632, (**c**, **d**) NSP6-ZINC-148,180,843, (**e**, **f**) NSP6-ZINC-089639800, (**g**, **h**) NSP6-ZINC-075486396, (**i**, **j**) NSP6-ZINC-157,610,683, (**k**, **l**) NSP6-ZINC-075484852, (**M**, **n**) NSP6-ZINC-016611776, and (**o**, **P**) NSP6-ZINC-141,457,420.

Molecular docking is a milestone process, from which we can predict the binding sites of the SARS-CoV-2 NSP6 protein, the binding energies, the binding scores, and the ligands with the highest potential. An energy minimization process was applied to the SARS-CoV-2 NSP6 protein then the binding sites were predicted by Autodock Vina as shown in Fig. 2. A library of all available lead-like ligands was downloaded from the ZINC20 database and was organized into a database of more than 2.9 million ligands using Autodock Vina. The ligand docking was applied using 20 docking runs for each ligand in the predicted binding sites. The ligands with the lowest binding energies were picked up, producing 8 ligands with the lowest binding energies (Table 4). From docking interactions, we found all ligands are found to bind with residues located in CTL and HTL epitopes. The docking interaction of the SARS-CoV-2 protein with the different 8 ligands is shown in Fig. 3.

**Table 4:**
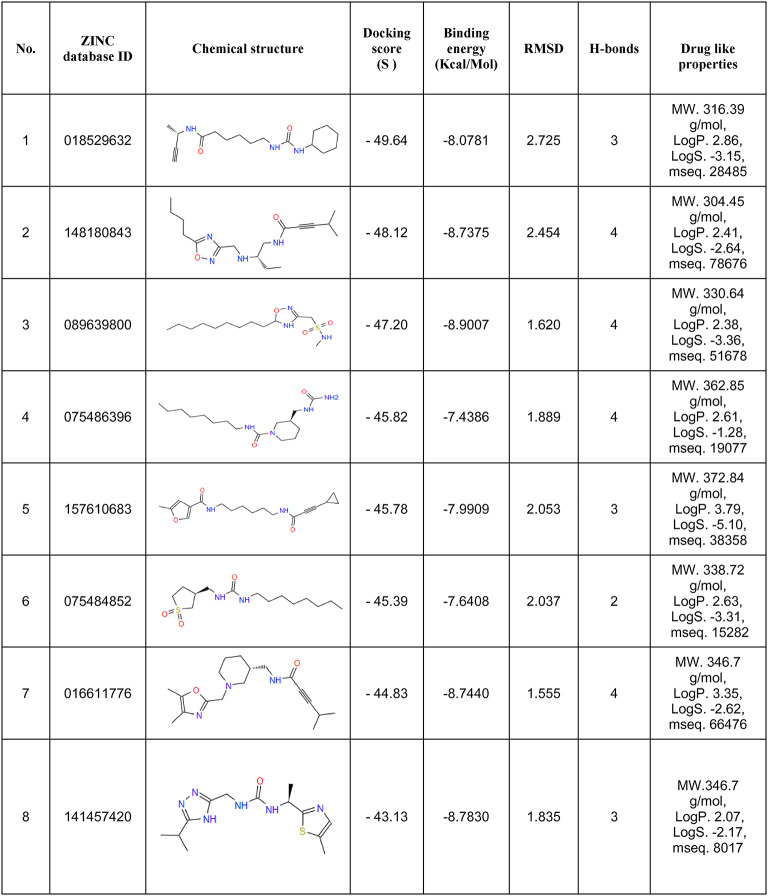
List of top-scored 8 drug-like molecules resulted from molecular Docking analyses using Autodock Vina.

### Physiochemical and drug-likeness analysis

To predict the favourable ADMET parameters of the selected ligands, we used the Swiss-ADME server, which used many parameters to assess the drug-likeness of the ligands. The molecular weight (MW) is used to figure out the draggability, the log P is a measurement tool of the hydrophilicity of the drug which indicates the absorption property, Lipiniski’s rule of five is used to test the bioavailability characteristics ADME (adsorption, distribution, metabolism, elimination) of the drug like molecule. All 8 ligands have MW less than 380 Da, and log P values are less than 5, and bioavailability score higher than 0.5, while only the ligand with ZINCID 00018529632, ZINCID 00148180843, ZINCID 00089639800, ZINCID 00157610683, ZINCID 00075484852, and ZINCID 00016611776 have no Lipiniski’s violations (Table 5).

**Table 5:** Drug-likeness properties of the selected drug like compounds obtained using the Swiss-ADME server.

	1	2	3	4	5	6	7	8
ZINC database ID	00018529632	00148180843	00089639800	00075486396	00157610683	00075484852	00016611776	00141457420
Molecular formula	C_18_H_24_N_2_O_3_	C_14_H_28_N_2_O_3_S	C_13_H_27_N_3_O_3_S	C_16_H_50_N-_4_O_2_	C_18_H_56_N_2_O_3_	C_14_H_34_N_2_O_3_S	C_18_H_55_N_3_O_2_	C_13_H_38_N_6_OS
Molecular weight (g/mol)	316.39	304.45	330.64	362.85	372.84	338.72	372.86	346.7
No. of H-bond acceptors	3	3	5	5	5	5	4	6
No. of H-bond donors	2	2	2	6	5	3	5	6
LogP (octanol/water partition coefficient)	2.86	2.41	2.38	2.61	3.79	2.63	3.35	2.07
Topological polar surface area	71.34	83.65	88.17	83.6	76.74	86.81	80.36	93.91
Lipinski no. of violations	0	0	0	1	0	0	0	1
Ghose no. of violations	0	0	1	2	2	2	2	2
Veber no. of violations	0	1	1	0	0	1	0	1
Bioavailability score	0.55	0.55	0.55	0.55	0.55	0.55	0.55	0.55
PAINS no. of alerts	0	0	0	0	0	0	0	0

### Toxicity prediction

**Fig. 4:**
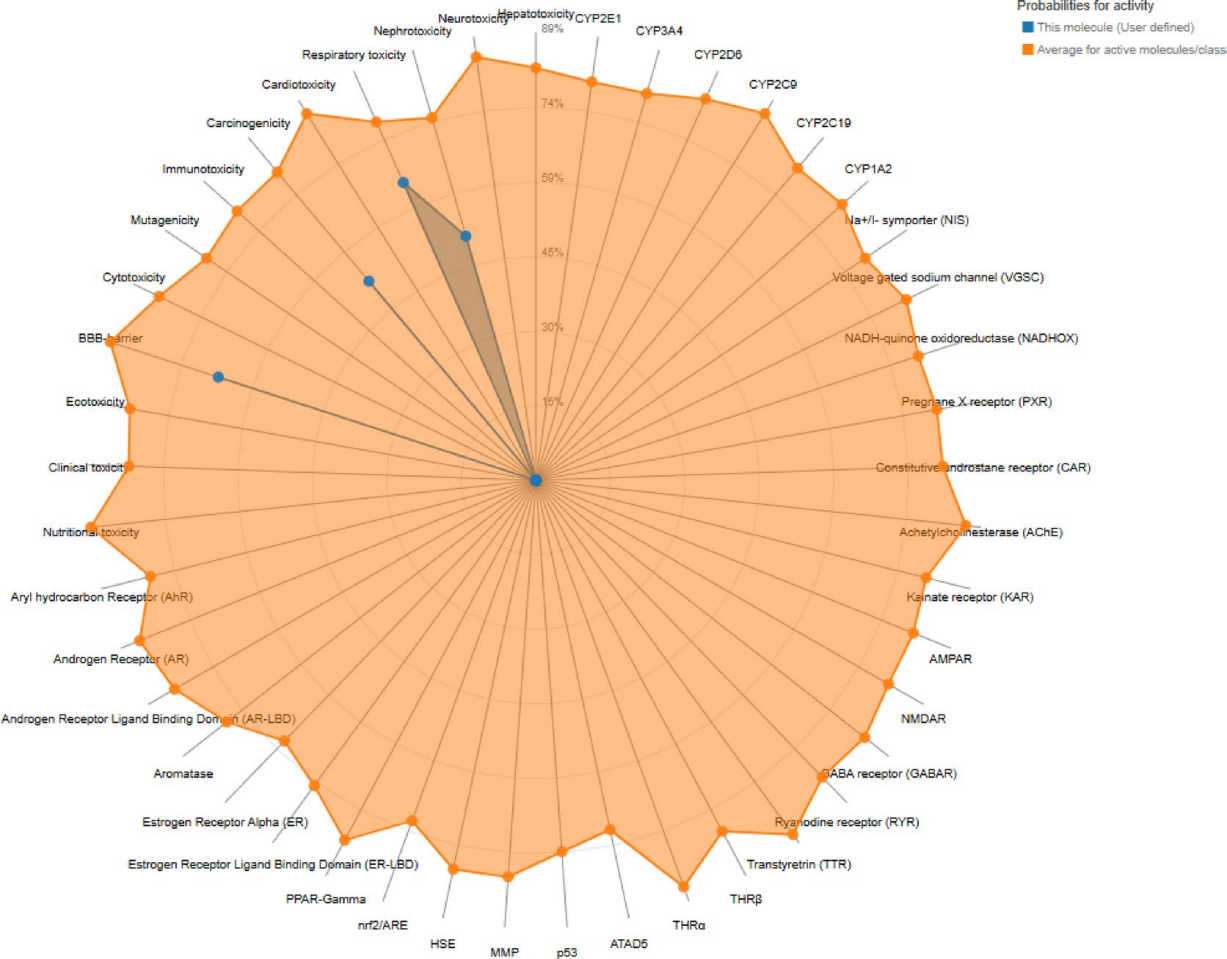
Toxicity prediction of the ligands using ProTox II server.

The ProTox III server was used to evaluate the toxicity of the selected 8 ligands, in which the ligands revealed no toxicity with cardiotoxicity, clinical toxicity, nephrotoxicity, carcinogenicity, mutagenicity, blood–brain barrier (BBB) penetration, cytotoxicity, or nutritional toxicity, as shown in (Fig. 4). ADMET-AI was used to predict the physicochemical, absorption, distribution, metabolism and excretion of the ligands in the body (Tables 6, 7, 8, 9, 10).

**Table 6:** Physicochemical properties of the selected drug like compounds obtained using the ADMET-AI server.

ZINC database ID	00018529632	00148180843	00089639800	00075486396	00157610683	00075484852	00016611776	00141457420	Units
Molecular weight	316.40	304.46	330.64	362.86	372.85	338.73	372.87	346.71	Dalton
LogP	2.41	2.08	4.75	7.31	6.86	5.37	7.86	5.31	log-ratio
Hydrogen bond acceptors	3.00	3.00	5.00	3.00	3.00	4.00	2.00	5.00	#
Hydrogen bond donors	2.00	2.00	2.00	4.00	3.00	3.00	3.00	6.00	#
Lipinski rule of 5	4.00	4.00	4.00	3.00	3.00	3.00	3.00	2.00	# of 4
Quantitative estimate of druglikeness (QED)	0.57	0.64	0.54	0.19	0.29	0.31	0.22	0.16	-
Stereo centers	0.00	1.00	1.00	0.00	1.00	1.00	0.00	1.00	#
Topological polar surface area (TPSA)	71.34	75.27	79.79	159.90	121.28	78.43	124.90	169.98	Å^2^

**Table 7:** Absorption properties of the selected drug like compounds obtained using the ADMET-AI server.

ZINC database ID	00018529632	00148180843	00089639800	00075486396	00157610683	00075484852	00016611776	00141457420	Units
Human intestinal absorption	1.00	0.99	1.00	1.00	1.00	1.00	1.00	1.00	-
Oral bioavailability	0.78	0.73	0.86	0.91	0.87	0.84	0.90	0.82	-
Aqueous solubility	-2.93	-2.83	-3.10	-3.38	-3.93	-4.16	-3.55	-3.13	log(mol/L)
Lipophilicity	3.17	2.04	1.99	1.39	1.68	2.05	1.66	1.56	log-ratio
Hydration free energy	-8.37	-8.82	-6.31	-5.68	-5.81	-7.53	-5.16	-7.53	kcal/mol
Cell effective permeability	-4.58	-4.80	-5.07	-5.52	-4.97	-5.18	-5.45	-5.68	log(10^− 6^ cm/s)
PAMPA permeability	0.96	0.74	0.92	0.88	0.82	0.89	0.90	0.85	-
P-glycoprotein inhibition	0.36	0.25	0.69	0.40	0.58	0.41	0.50	0.46	-

**Table 8:** Distribution properties of the selected drug like compounds obtained using the ADMET-AI server.

ZINC database ID	00018529632	00148180843	00089639800	00075486396	00157610683	00075484852	00016611776	00141457420	Units
Blood-brain barrier penetration	0.84	0.97	0.99	0.99	0.96	0.98	0.99	0.94	-
Plasma protein binding rate	98.99	75.05	92.06	79.40	79.36	81.11	83.42	78.64	%
Volume of distribution at steady state	1.86	2.34	4.64	12.94	25.06	14.26	14.11	7.90	L/kg

**Table 9:** Metabolism properties of the selected drug like compounds obtained using the ADMET-AI server.

ZINC database ID	00018529632	00148180843	00089639800	00075486396	00157610683	00075484852	00016611776	00141457420	Units
CYP1A2 inhibition	0.90	0.05	0.03	0.05	0.11	0.11	0.06	0.07	-
CYP2C19 inhibition	0.92	0.15	0.45	0.14	0.06	0.24	0.13	0.47	-
CYP2C9 inhibition	0.70	0.05	0.28	0.05	0.03	0.17	0.04	0.22	-
CYP2D6 inhibition	0.25	0.02	0.18	0.32	0.19	0.15	0.36	0.31	-
CYP3A4 inhibition	0.75	0.18	0.16	0.05	0.02	0.03	0.05	0.17	-
CYP2C9 substrate	0.34	0.44	0.27	0.11	0.06	0.21	0.12	0.16	-
CYP2D6 substrate	0.29	0.41	0.22	0.55	0.48	0.26	0.55	0.43	-
CYP3A4 substrate	0.68	0.57	0.70	0.54	0.38	0.59	0.56	0.61	-

**Table 10:** Excretion properties of the selected drug like compounds obtained using the ADMET-AI server.

ZINC database ID	00018529632	00148180843	00089639800	00075486396	00157610683	00075484852	00016611776	00141457420	Units
Half life	26.06	28.03	36.45	54.12	65.12	20.86	56.84	51.97	hr
Drug clearance (Hepatocyte)	70.98	55.65	69.06	40.31	31.96	56.31	47.87	36.25	uL/min/10^6^ cells
Drug clearance (Microsome)	31.45	19.01	24.19	14.26	12.40	35.34	20.19	26.47	uL/min/mg

### Molecular dynamics simulations

The simulation was applied to the unbounded NSP6 (apo) and the NSP6-ligand complexes at a 100 ns time function. The changes in the trajectories and parameters of NSP6-Ligand complexes compared to NSP6-APO were analysed according to different parameters, which are Root Mean Square Deviations (RMSDs), gyration radius, Root Mean Square Fluctuations (RMSFs), and intermolecular hydrogen bond formation.

### RMSD

**Fig. 5:**
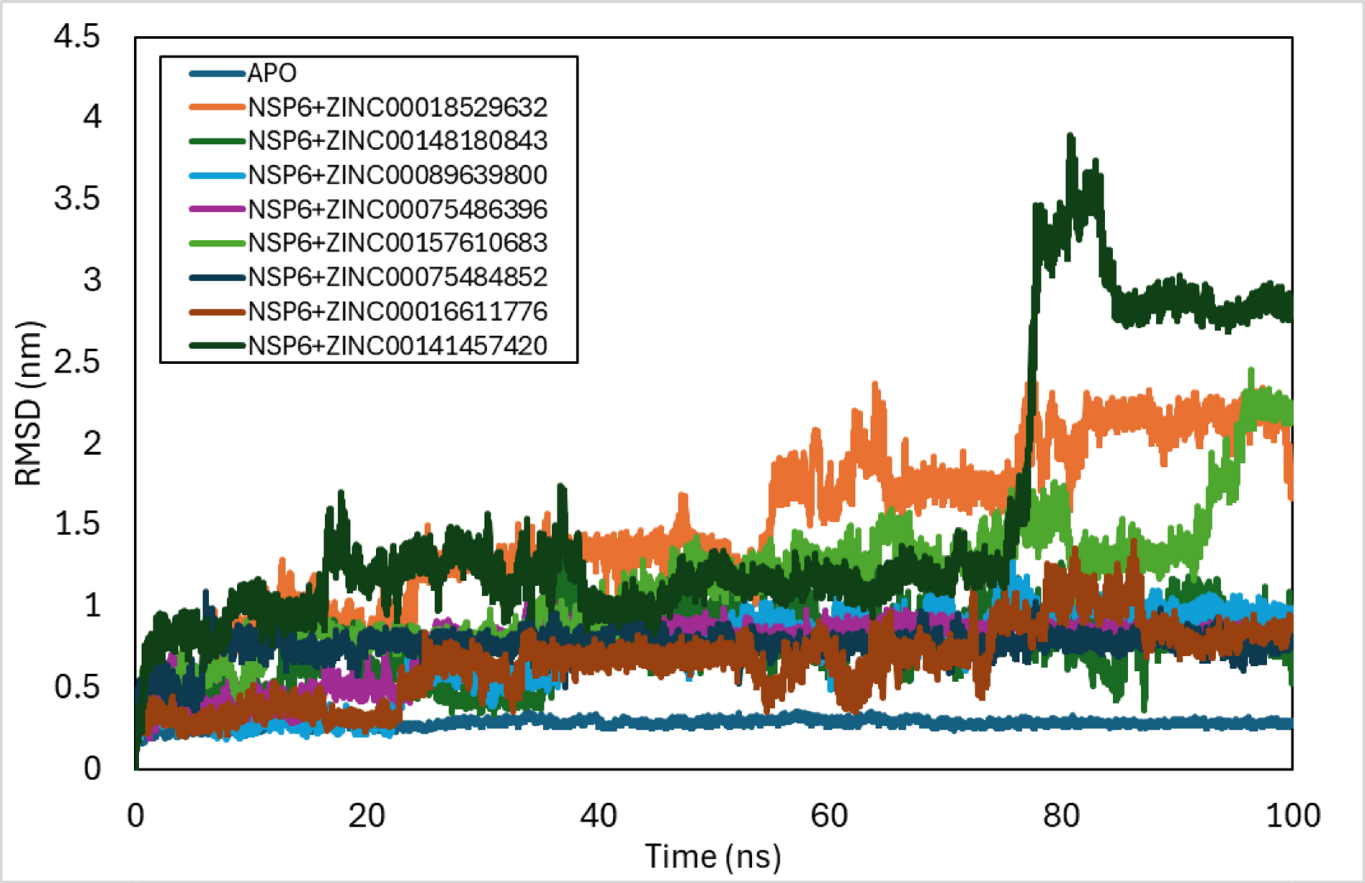
RMSD plot of docked complexes generated through MDS at 100 ns.

The structural and conformational stability of the SARS-CoV-2 protein docked with the selected ZINC complexes was estimated by evaluating the RMSD of the protein backbone atoms against the time of simulation (Fig. 5). The RMSD analysis of various NSP6 complexes revealed distinct stability patterns over a 100 ns simulation. The NSP6-APO complex exhibited a sharp increase in RMSD up to 20 ns, stabilizing between 0.2 and 0.35 nm. The NSP6-ZINC-018529632 complex provided stability in four distinct periods, with RMSD ranging from 0.5 to 2.3 nm. The NSP6-ZINC-148,180,843 complex demonstrated fluctuating RMSD values between 0.3 and 0.8 nm, with increased fluctuations after 35 ns. The NSP6-ZINC-089639800 complex stabilized after 80 ns, maintaining RMSD values between 0.8 and 1 nm. The NSP6-ZINC-075486396 complex reached stability at 35 ns with a decreasing RMSD trend from 1 nm to 0.75 nm. The NSP6-ZINC-157,610,683 complex was briefly stable between 11 and 37 ns before a continuous RMSD increase to 2.2 nm by 100 ns. The NSP6-ZINC-075484852 complex achieved early stability at 10 ns, maintaining RMSD around 0.75 nm with minor peaks. The NSP6-ZINC-016611776 complex was stable only between 33 and 50 ns, with instability thereafter. Finally, the NSP6-ZINC-141,457,420 complex showed stability from 38 to 75 ns with RMSD at 1.3 nm before a significant increase to 2.8 nm by the end of the simulation.

### RMSF

**Fig. 6:**
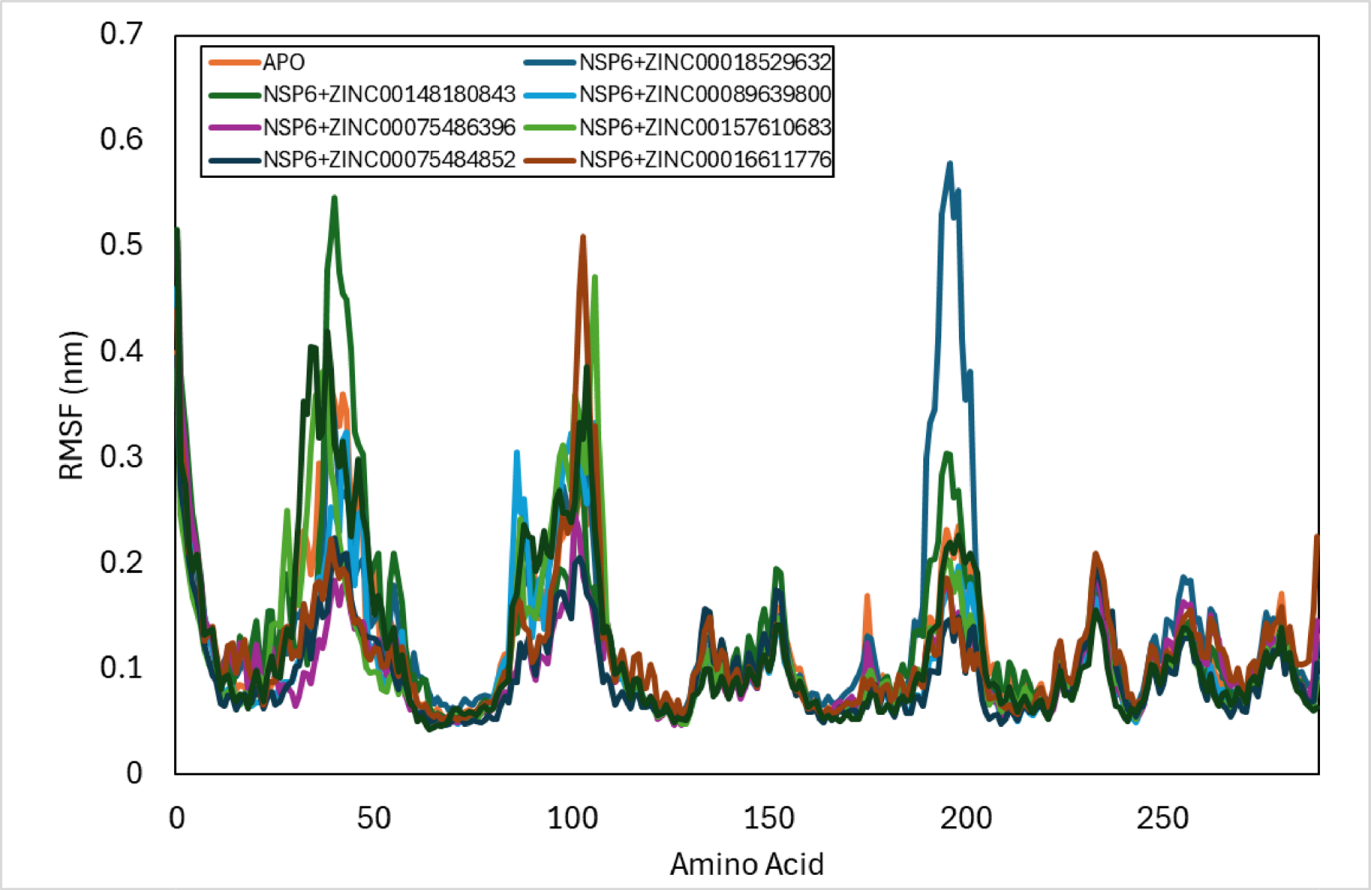
RMSF plot of docked complexes generated through MDS at 100 ns.

RMSF is the average estimation of the displacement of a specific group of atoms or a structure to a reference structure. It is important in the prediction of the flexibility of the residues and the protein backbone. The RMSF analysis of the NSP6-ligands complexes is calculated along the timescale of the simulation 100 ns (Fig. 6). The average RMSF values for NSP6 complexes with ZINC compounds were analysed over a 100 ns simulation (Table 11), focusing on the residue regions 30:50, 80:100, and 170:200. Compared to the NSP6-apo, the NSP6-ZINC-018529632 complex exhibited lower fluctuations in the 30:50 region, while higher fluctuations in the 80:100 and 170:200 regions. The NSP6-ZINC-148,180,843 complex showed higher fluctuations in the 30:50 and 170:200 regions and lower in the 80:100 region. The NSP6-ZINC-089639800 and NSP6-ZINC-016611776 complexes both had lower fluctuations in the 30:50 and 170:200 regions and higher in the 80:100 region. The NSP6-ZINC-075486396 and NSP6-ZINC-075484852 complexes displayed consistently lower fluctuations across all three regions. The NSP6-ZINC-157,610,683 complex had a leftward shift in the 30:50 region and exhibited higher fluctuations in the 80:100 region, but lower in the 170:200 region. Finally, the NSP6-ZINC-141,457,420 complex showed higher fluctuations in the 30:50 and 80:100 regions, with no difference in the 170:200 region compared to the NSP6-apo.

**Table 11:** The average RMSF values for NSP6 complexes with ZINC compounds.

Complex	Average RMSF (nm)
NSP6 + 00018529632	0.14 ± 0.09
NSP6 + 00148180843	0.13 ± 0.08
NSP6 + 00089639800	0.11 ± 0.07
NSP6 + 00075486396	0.10 ± 0.05
NSP6 + 00157610683	0.12 ± 0.07
NSP6 + 00075484852	0.10 ± 0.05
NSP6 + 00016611776	0.12 ± 0.06
NSP6 + 00141457420	0.12 ± 0.08

### Radius of gyration

**Fig. 7:**
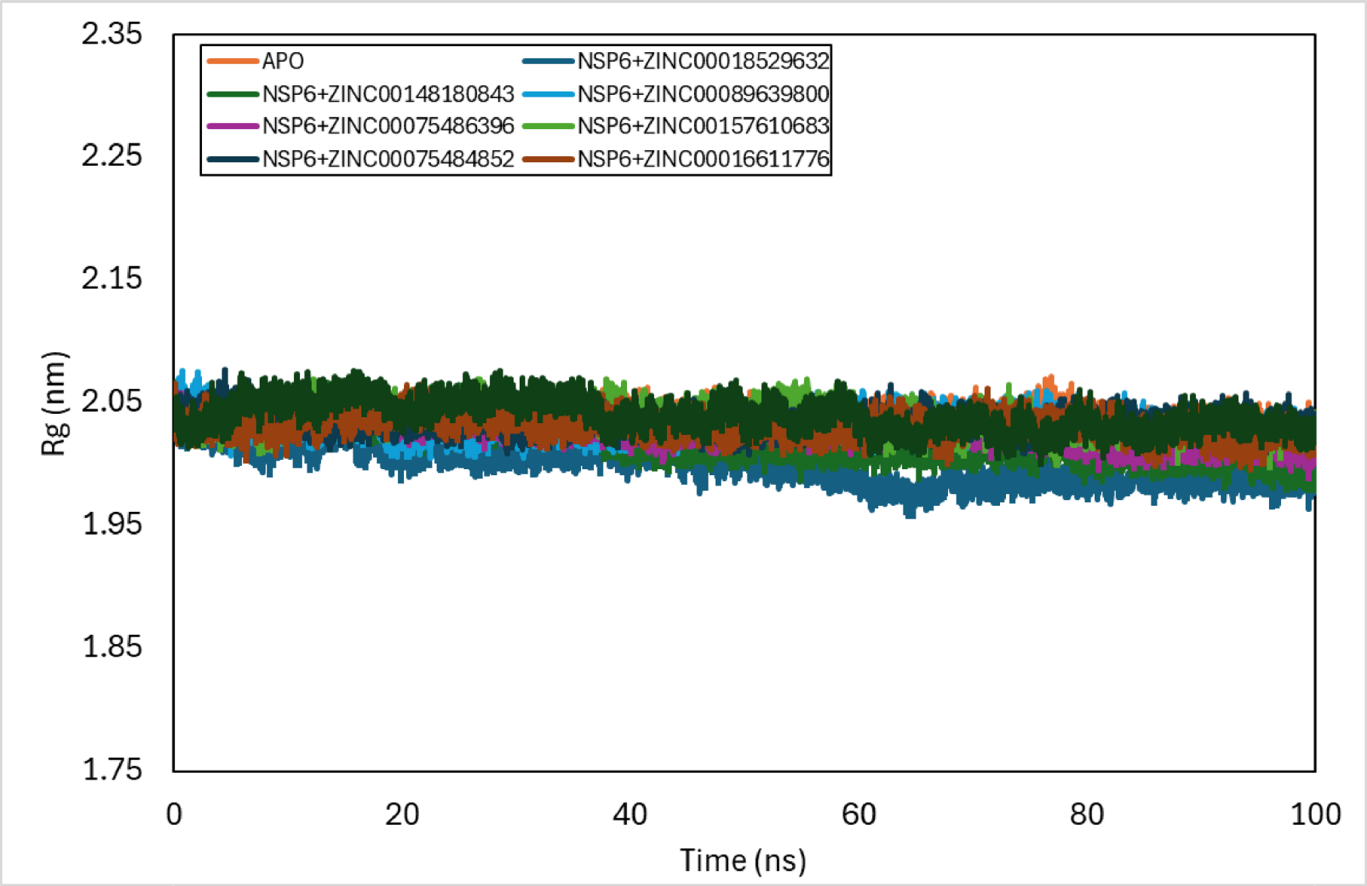
Rg plot of docked complexes generated through MDS at 100 ns.

Radius of gyration (Rg) is an important parameter in the investigation of the compactness and integrity of the complex’s structure which is used in the evaluation of the stability of the system. It is the mass-weighted RMSD of atoms from their centre of mass. The average Rg values of NSP6-ZINC-018529632, NSP6-ZINC-148,180,843, NSP6-ZINC-089639800, NSP6-ZINC-075486396, NSP6-ZINC-157,610,683, NSP6-ZINC-075484852, NSP6-ZINC-016611776, and NSP6-ZINC-141,457,420 complexes from 0 to 100 ns were 2.00 ± 0.02 nm, 2.02 ± 0.02 nm, 2.03 ± 0.01 nm, 2.03 ± 0.01 nm, 2.04 ± 0.01 nm, 2.03 ± 0.01 nm, 2.03 ± 0.01 nm, and 2.04 ± 0.01 nm, respectively as shown in Fig. 7.

### SASA analysis

**Fig. 8:**
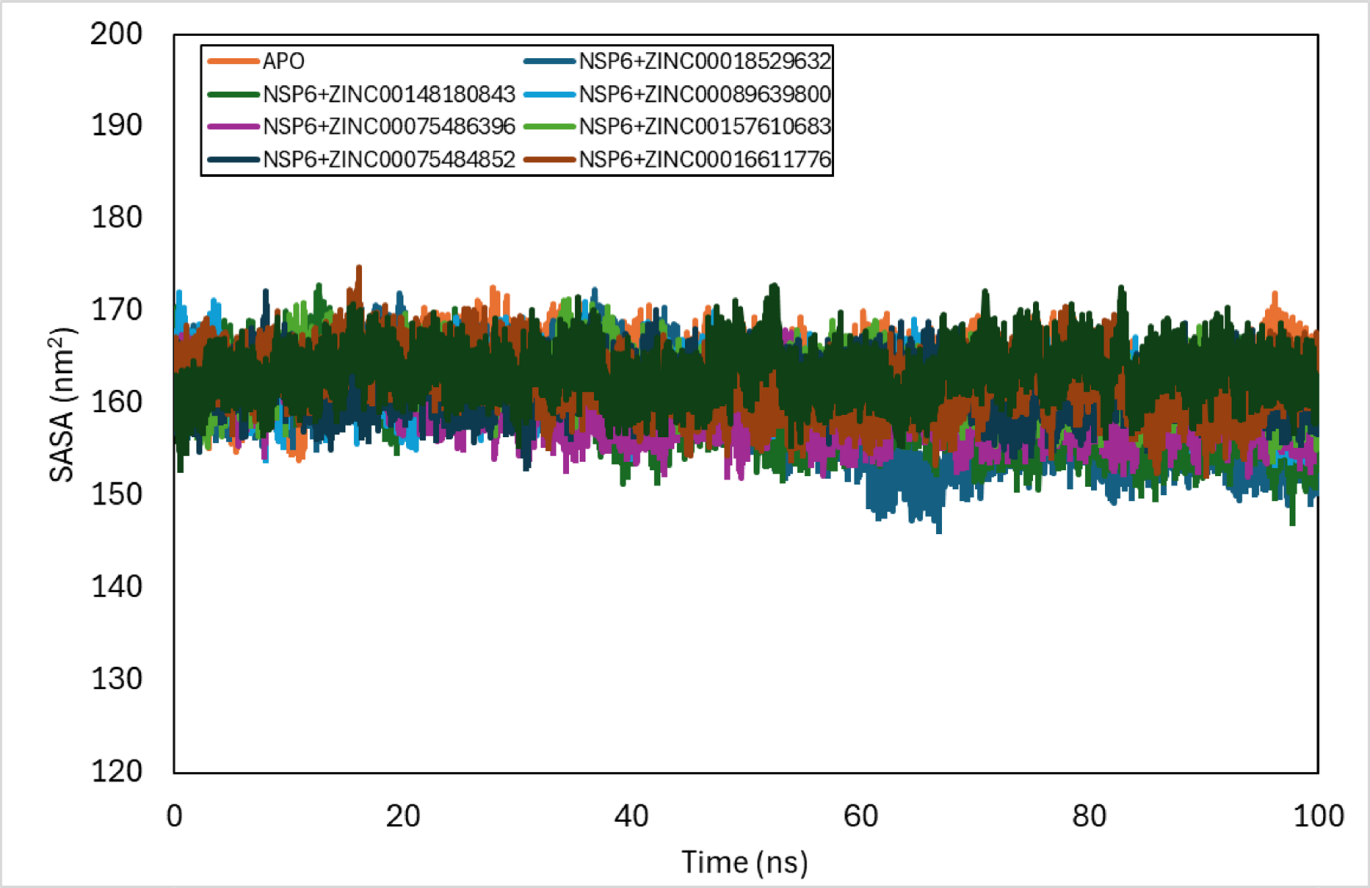
SASA plot of docked complexes generated through MDS at 100 ns.

To have precise knowledge about the complex stability, folding, and compactness of the hydrophobic core, the solvent-accessible surface area (SASA) was used to estimate the exposed area of the complex that interacts with the surrounding solvent molecules. The changes in the SASA for the complexes along the simulation period (from 0 to 100 ns) were analysed (Fig. 8). The average SASA values for the NSP6-ZINC-018529632, NSP6-ZINC-148,180,843, NSP6-ZINC-089639800, NSP6-ZINC-075486396, NSP6-ZINC-157,610,683, NSP6-ZINC-075484852, NSP6-ZINC-016611776, and NSP6-ZINC-141,457,420 complexes from 0 to 100 ns were 159.3 ± 4.83 nm^2^, 159.5 ± 3.68 nm^2^, 162.0 ± 2.49 nm^2^, 159.6 ± 2.53 nm^2^, 162.5 ± 2.49 nm^2^, 161.9 ± 2.27 nm^2^, 162.3 ± 2.86 nm^2^, and 163.4 ± 2.49 nm^2^, respectively.

### Molecular interactions analysis

**Fig. 9:**
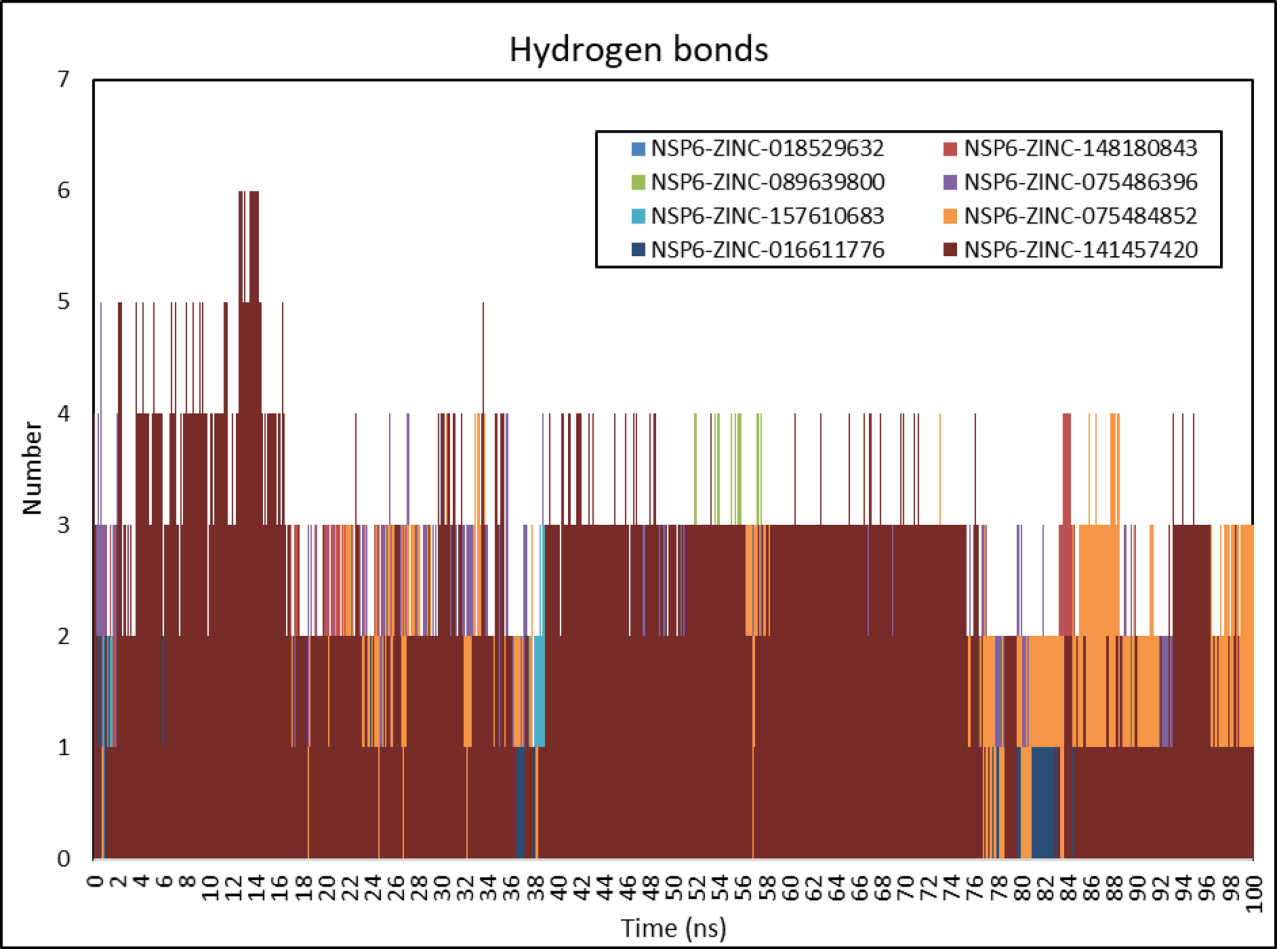
H-bond analysis of docked complexes generated through MDS at 100 ns. (a) NSP6-ZINC-018529632, (b) NSP6-ZINC-148,180,843, (c) NSP6-ZINC-089639800, (d) NSP6-ZINC-075486396, (e) NSP6-ZINC-157,610,683, (f) NSP6-ZINC-075484852, (g) NSP6-ZINC-016611776, and (h) NSP6-ZINC-141,457,420.

The hydrogen bonds between the protein and ligand are important in maintaining the stability of the complex. CHAARM force field was used to investigate the hydrogen bonds in the NSP6-ligands complexes. The estimation of H-bonds of the NSP6-ZINC-018529632, NSP6-ZINC-148,180,843, NSP6-ZINC-089639800, NSP6-ZINC-075486396, NSP6-ZINC-157,610,683, NSP6-ZINC-075484852, NSP6-ZINC-016611776, and NSP6-ZINC-141,457,420 complexes are from 0 to 100 ns. The NSP6-ZINC-141,457,420 complex has the highest H-bonds with 16,278 H-bonds, followed by NSP6-ZINC-075486396 with 16,234 H-bonds, and the lowest was NSP6-ZINC-016611776 with 2964 H-bonds. The maximum number of H-bonds was six bonds in NSP6-ZINC-141,457,420 complex (Fig. 9).

### MM-PBSA analysis

Analysing the physical energies between the ligand and the NSP6 protein could increase the certainty of predicting the best ligand-protein complex. The MMPBSA analysis was used to predict the best pose of docking of the ligand-protein complex following the MD simulations. MMPBSA can differentiate the significant differences in the complexes and focus on the protein’s active sites, which results in the energies of the stability of the complexes. The MMPBSA was used to analyse the binding energies of NSP6-ZINC-018529632, NSP6-ZINC-148,180,843, NSP6-ZINC-089639800, NSP6-ZINC-075486396, NSP6-ZINC-157,610,683, NSP6-ZINC-075484852, NSP6-ZINC-016611776, and NSP6-ZINC-141,457,420 complexes. The various types of energies per each complex such as Van der Waals, Electrostatic, Polar Solvation, and Binding energy are presented in Table 12. Van der Waals interactions play a higher significant effect than the electrostatic contacts in the analysis of the binding energies in all complexes except in NSP6-ZINC-141,457,420 where the electrostatic contacts were more effective than the Van der Waals with respective energies of -25.33 ± 15.22 and − 24.64 ± 5.65 kcal/Mol. The higher Van der Waals energy indicates the higher attractive forces between the molecules while the higher electrostatic contacts indicate a higher repulsive force. While the positive polar solvation energy indicates the amount of gained energy by the system when immersed in a polar solvent. The magnitude of the binding energy indicates the strength of the interactions between the ligand and the NSP6.

**Fig. 10:**
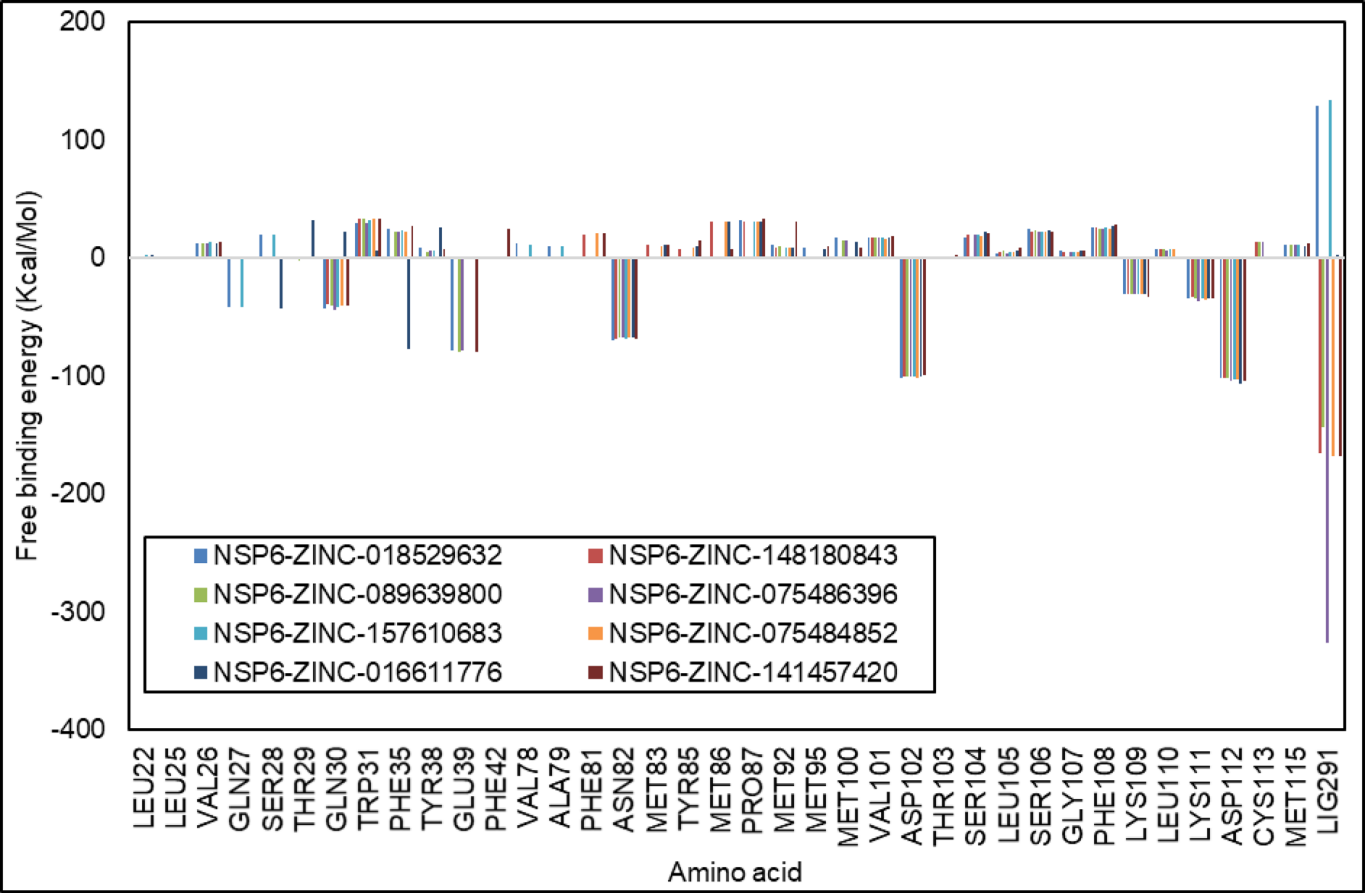
Binding free energy decomposition of NSP6-ZINC-018529632, NSP6-ZINC-148,180,843, NSP6-ZINC-089639800, NSP6-ZINC-075486396, NSP6-ZINC-157,610,683, NSP6-ZINC-075484852, NSP6-ZINC-016611776, and NSP6-ZINC-141,457,420.

Through applying the decomposition analysis, the free energy situation of individual amino acids in the complexes within 1 nm is analysed for the NSP6-ZINC-018529632, NSP6-ZINC-148,180,843, NSP6-ZINC-089639800, NSP6-ZINC-075486396, NSP6-ZINC-157,610,683, NSP6-ZINC-075484852, NSP6-ZINC-016611776, and NSP6-ZINC-141,457,420 complexes (Fig. 10).

**Table 12:** The Estimation of various energy observed in NSP6 + ligands systems.

Complex	Van der Waals energy (kcal/Mol)	Electrostatic energy (kcal/Mol)	Polar solvation energy (kcal/Mol)	Binding energy (kcal/Mol)
NSP6-ZINC-018529632	-39.08 ± 4.27	-18.16 ± 7.14	24.46 ± 5.77	-32.78 ± 5.31
NSP6-ZINC-148,180,843	-31.87 ± 4.03	-9.53 ± 10.23	17.14 ± 9.51	-24.26 ± 3.92
NSP6-ZINC-089639800	-34.77 ± 4.85	-11.73 ± 7.54	22.93 ± 5.87	-23.57 ± 4.55
NSP6-ZINC-075486396	-32.28 ± 4.23	-22.58 ± 9.18	25.48 ± 7.4	-29.39 ± 5.77
NSP6-ZINC-157,610,683	-31.81 ± 5.69	-6.48 ± 8.00	13.21 ± 7.86	-25.08 ± 4.67
NSP6-ZINC-075484852	-38.78 ± 5.59	-10.49 ± 9.48	18.4 ± 8.48	-30.87 ± 6.26
NSP6-ZINC-016611776	-28.97 ± 4.23	-10.66 ± 7.6	16.77 ± 6.15	-22.86 ± 4.44
NSP6-ZINC-141,457,420	-24.64 ± 5.65	-25.33 ± 15.22	29.8 ± 14.33	-20.17 ± 6.34

## Discussion

NSP6 is one of the most attractive SARS-CoV-2 NSPs for drug targeting, though it does not have a verified crystal structure till now which allows the researchers to use bioinformatical tools to predict its tertiary structure model. This study aims to find the most possible vaccine against SARS-CoV2 NSP6 protein.

The tertiary structure model of the NSP6 was generated using the Alphafold server, and its quality was evaluated using The SAVES server, and the PROCHECK server and the quality percent were 99.94% and 94.4% of the structure was in the core region with 5.6% in the allowed region of the Ramachandran plot, which indicates the quality of the predicted model. The possible epitopes of the NSP6 were predicted and resulted in 3 CTL epitopes, 26 HTL epitopes, and 5 B-cell epitopes.

To detect the most suitable ligands candidates to target the NSP6 protein, first the full library of ligands will be docked with NSP6, then the selected docked group will be subjected to MDS study to evaluate their interactions with the protein through RMSD, RMSF, SASA, Rg, H-bonds, and free energy studies.A full library of suitable lead-like ligands was downloaded from the ZINC20 database, these ligands were visualized and docked against NSP6 on different 20 runs for each ligand, producing 8 ligands with the lowest available binding energies and RMSD. The Physiochemical and Drug-Likeness of these ligands were studied, in accordance with the Lipinski’s rule of 5, all 8 ligands have MW less than 380 Da, and log P values are less than 5, and bioavailability score higher than 0.5, while ZINCID 00018529632, ZINCID 00148180843, ZINCID 00089639800, ZINCID 00157610683, ZINCID 00075484852, and ZINCID 00016611776 ligands with have no Lipinski’s violations. ZINCID 00018529632 showed that it has no Lipinski, Ghose and Veber violations, also it has no PAINS alerts, and its bioavailability score is 0.55, however, it displayed the lowest H-bond acceptors or donors with a moderate molecular weight, which indicates the best availability and performance.

The MD simulations of the NSP6 complexes with the 8 ligands were run at 100 ns time function to study the changes in the trajectories and the parameters of the NSP6 complexes compared to NSP6-APO. The RMSD of the NSP6-APO complex increased sharply up to 20 ns before settling between 0.2 and 0.35 nm. Four different times, the NSP6-ZINC-018529632 compound demonstrated stability, with an RMSD ranging from 0.5 to 2.3 nm. The RMSD values of the NSP6-ZINC-148,180,843 complex fluctuated between 0.3 and 0.8 nm, and after 35 ns, the variations became larger. After 80 ns, the NSP6-ZINC-089639800 complex stabilized, keeping its RMSD values within the range of 0.8 and 1 nm. The RMSD trend of the NSP6-ZINC-075486396 complex decreased from 1 nm to 0.75 nm before reaching stability at 35 ns. The RMSD of the NSP6-ZINC-157,610,683 complex increased continuously to 2.2 nm by 100 ns after a brief period of stability between 11 and 37 ns. Early stability was attained by the NSP6-ZINC-075484852 complex at 10 ns, with minimal peaks and an RMSD of about 0.75 nm. Only from 33 to 50 ns did the NSP6-ZINC-016611776 complex exhibit stability before becoming unstable. Before experiencing a notable increase to 2.8 nm by the end of the simulation, the NSP6-ZINC-141,457,420 complex demonstrated stability from 38 to 75 ns with RMSD at 1.3 nm.

The average RMSF values for NSP6 complexes with ZINC compounds were analysed over a 100 ns simulation, the NSP6-ZINC-00075486396 and NSP6-ZINC-00075484852 have the lowest average RMSF value 0.10 ± 0.05 nm, while the value of NSP6-ZINC-00089639800 is 0.11 ± 0.07 nm, more over the NSP6-ZINC-00157610683, NSP6-ZINC-00016611776, and NSP6-ZINC-00141457420 values are 0.12 ± 0.07, 0.12 ± 0.06, and 0.12 ± 0.08 nm, respectively. In addition, the value of NSP6-ZINC-00148180843 is 0.13 ± 0.08 nm and the value of NSP6-ZINC-00018529632 is 0.14 ± 0.09 nm. This indicates that the NSP6-ZINC-00075486396 and NSP6-ZINC-00075484852 complexes have the highest stability.

The average Radius of gyration values of NSP6-ZINC-018529632, NSP6-ZINC-148,180,843, NSP6-ZINC-089639800, NSP6-ZINC-075486396, NSP6-ZINC-157,610,683, NSP6-ZINC-075484852, NSP6-ZINC-016611776, and NSP6-ZINC-141,457,420 complexes from 0 to 100 ns were 2.00 ± 0.02 nm, 2.02 ± 0.02 nm, 2.03 ± 0.01 nm, 2.03 ± 0.01 nm, 2.04 ± 0.01 nm, 2.03 ± 0.01 nm, 2.03 ± 0.01 nm, and 2.04 ± 0.01 nm, respectively. While the NSP6-ZINC-018529632 complex revealed a decreasing trend starting at 20 ns to 60 ns then it reached stability till 100 ns at 1.97 nm, the NSP6-ZINC-148,180,843 complex exhibited a decrease at 40 ns and remained stable to 100 ns with Rg value of 2 nm, and NSP6-ZINC-075486396 showed a decreasing trend starting at the 70 ns to the 100 ns at 2 nm. The NSP6-ZINC-089639800, NSP6-ZINC-157,610,683, NSP6-ZINC-075484852, NSP6-ZINC-016611776, and NSP6-ZINC-141,457,420 complexes exhibited minimal deviation from the NSP6-apo protein, indicating that their binding does not significantly alter the protein’s structural stability or dynamics.

The changes in the solvent-accessible surface area for the complexes along the simulation period (from 0 to 100 ns) were analysed. The average SASA values for the NSP6-ZINC-018529632, NSP6-ZINC-148,180,843, NSP6-ZINC-089639800, NSP6-ZINC-075486396, NSP6-ZINC-157,610,683, NSP6-ZINC-075484852, NSP6-ZINC-016611776, and NSP6-ZINC-141,457,420 complexes from 0 to 100 ns were 159.3 ± 4.83 nm^2^, 159.5 ± 3.68 nm^2^, 162.0 ± 2.49 nm^2^, 159.6 ± 2.53 nm^2^, 162.5 ± 2.49 nm^2^, 161.9 ± 2.27 nm^2^, 162.3 ± 2.86 nm^2^, and 163.4 ± 2.49 nm^2^, respectively. The NSP6-ZINC-089639800, NSP6-ZINC-157,610,683, NSP6-ZINC-075484852, NSP6-ZINC-016611776, and NSP6-ZINC-141,457,420 complexes exhibited stability similar to the NSP6-apo protein, in contrast, the NSP6-ZINC-018529632 complex showed a decline in activity up to 60 ns but stabilized until 100 ns. Similarly, the NSP6-ZINC-148,180,843 complex experienced a decrease at 40 ns, remaining stable afterward. Lastly, the NSP6-ZINC-075486396 complex revealed a decrease at the 20ns and then remained stable.

The estimation of H-bonds of the NSP6-ZINC-018529632, NSP6-ZINC-148,180,843, NSP6-ZINC-089639800, NSP6-ZINC-075486396, NSP6-ZINC-157,610,683, NSP6-ZINC-075484852, NSP6-ZINC-016611776, and NSP6-ZINC-141,457,420 complexes from 0 to 100 ns, indicates that the NSP6-ZINC-141,457,420 complex has the highest H-bonds with 16,278 H-bonds, followed by NSP6-ZINC-075486396 with 16,234 H-bonds, and the lowest was NSP6-ZINC-016611776 with 2964 H-bonds. The maximum number of H-bonds was six bonds in the NSP6-ZINC-141,457,420 complex, which suggests that it has the highest binding affinity.

The MMPBSA was used to analyse the binding energies of NSP6-ZINC-018529632, NSP6-ZINC-148,180,843, NSP6-ZINC-089639800, NSP6-ZINC-075486396, NSP6-ZINC-157,610,683, NSP6-ZINC-075484852, NSP6-ZINC-016611776, and NSP6-ZINC-141,457,420 complexes. Van der Waals interactions play a higher significant effect than the electrostatic contacts in the analysis of the binding energies in all complexes except in NSP6-ZINC-141,457,420 where the electrostatic contacts were more effective than the Van der Waals with respective energies of -25.33 ± 15.22 and − 24.64 ± 5.65 kcal/Mol. The magnitude of the binding energy indicates the strength of the interactions between the ligand and the NSP6. NSP6-ZINC-018529632 complex was found to have the lowest binding energy score with − 32.78 ± 5.31 kcal/Mol indicating the best stability among the other complexes.

Due to their remarkable results in the RMSD, RMSF, Rg, SASA, H-bonds, and MMPBSA, ZINC-141,457,420, ZINC-075486396, and ZINC-018529632 ligands indicate a promising potential to be developed as potential vaccines against SARS-CoV-2 NSP6 protein.

Using the same sequence of study, Ahmed Abdelkader and colleagues studied the suitability of using FDA-approved drugs (DrugBank), Northern African Natural Products Database (NANPDB) and South African Natural Compounds Database (SANCDB) products, they have used the same tools and methods we used in our study in the tertiary structure prediction, visualization and docking, and the MD simulations for 100 ns. But, for the energy estimations they used MMGBSA, while we used the MMPBSA which is slower and more expensive in resources but reveals more accurate results^17^. All our 8 ligands are found to have better pharmacokinetics results than their 9 potential candidates. Also, only one complex only from their candidates is found to have lower binding energy than our ligands. Their candidates are found to likely bind more with residues VAL241, LY263, LYS285, ALA287, THR288, and GLN290, and LYS8, MET85, and TRP13, while our ligands are found to bind mainly with GLN30 and LYS109, and most of them can bind easily with LEU105, SER106, ASP102, PHE35, VAL101, and THR29 (B, and T-cell epitopes) which suggests the more suitability of our ligands.

The eight identified ligands, particularly ZINC-141,457,420, ZINC-075486396, and ZINC-018529632, exhibit promising drug-like properties, including compliance with Lipinski’s rule of five, high bioavailability scores, and no predicted toxicity (e.g., cardiotoxicity, nephrotoxicity, or carcinogenicity) as assessed by the Swiss-ADME and ProTox III servers. These characteristics suggest their potential for repurpose as inhibitors of SARS-CoV-2 NSP6, a critical protein in viral replication and transcription. The strong binding affinities, with docking scores ranging from − 49.64 to -43.13 kcal/mol and stable interactions observed in molecular dynamics simulations (e.g., high H-bond formation in NSP6-ZINC-141457420 and NSP6-ZINC-075486396 complexes), further support their suitability for repurposing. These compounds could be prioritized for experimental validation to assess their efficacy in inhibiting SARS-CoV-2 replication, potentially offering a rapid pathway to therapeutic development for COVID-19^18^.

Therefore, there is a requirement for more in vitro and in vivo tests so that the safety, efficacy, and toxicity of the vaccine and drug products can be confirmed. The study has pointed out how medicinal remedies may be designed using bioinformatics, virtual screening, and experimental validation.

## Conclusion

In response to the ongoing COVID-19 pandemic, significant efforts have been directed toward identifying potential therapeutic agents inhibiting SARS-CoV-2 NSP6 protein. Due to the lack of presence of a crystal structure of NSP6, a high-quality refined tertiary structure model of the SARS-CoV-2 NSP6 protein was predicted using AlphaFold. Structure-based virtual screening was performed using AutoDock Vina for ZINC20 library of ligands to dock with NSP6. Molecular dynamics studies were performed individually for Protein-ligand complexes at 100 ns. The binding stability and conformational dynamics of the protein-ligand complexes were analysed to assess the potential effectiveness of the drug-receptor interactions within a biological context. Based on comparative analyses of RMSD, RMSF, radius of gyration (Rg), and (SASA) across eight protein-ligand complexes, the NSP6-ZINC-089639800, NSP6-ZINC-157,610,683, NSP6-ZINC-075484852, NSP6-ZINC-016611776, and NSP6-ZINC-141,457,420 complexes exhibited minimal deviation from the NSP6 apo-protein in Rg and SASA profiles, while demonstrating favourable stability and flexibility in RMSD and RMSF analyses. The MM-PBSA analysis of the complexes showed that NSP6-ZINC-018529632 complex was found to have the lowest binding energy score indicating the best stability among the other complexes. Therefore, the ZINC-141,457,420, ZINC-075486396, and ZINC-018529632 ligands provided a promising potential that can be developed as potential vaccines inhibiting SARS-CoV-2 NSP6 protein.

## Methods

### Structure modelling of NSP6

The SARS-CoV-2 NSP6 protein is missing a confirmed 3-D structure, hence numerous research has been conducted utilizing various servers to estimate its tertiary structure^7^. The secondary structure of the protein was predicted using PSIPRED server^19^. To determine the tertiary structure of the SARS-CoV-2 NSP6 protein, we employed the Alphafold service. Alphafold is a server that predicts the tertiary structure of proteins using deep neural network learning methods via DeepMind algorithms. Version 3, which was made available in August 2020, is the most recent version currently in use. Atomic collisions, the poor backbone dihedral angles, and the MolProbity score all indicate better prediction quality^20–22^. Using the DeepRefiner server, the upcoming Alphafold model is refined to provide a higher-quality model^23^, and then the refined model was evaluated using SAVES server to validate the protein structure^24^.

### Prediction of cytotoxic T lymphocytes epitope

Cytotoxic T lymphocyte (CTL) epitope prediction is crucial for detecting the vaccine. The NetCTL 1.2 server was utilized for analyzing the amino acid sequence to anticipate the CTL epitopes^25^. First, the MHC-I binding affinity; second, the proteasomal C terminal cleavage carried out by artificial neural networks (ANN); and third, the weight matrix-predicted TAP (Transporter Associated with Antigen Processing) transport efficiency—are the three main factors that determine the epitopes. The thresholds for TAP transport efficiency, proteasomal C-terminal cleavage, and epitope identification were chosen at 0.05, 0.15, and 0.75, respectively, to predict the CTL epitopes. The aggregate score was used to categorize the predicted epitopes^26^.

### Prediction of Helper T lymphocytes epitope

Helper T lymphocytes (HTL) epitope prediction has been accomplished using the IEDB MHC II server^27^. The species/locus was chosen as Human/HLA-DR, and a 7-allele human leukocyte antigen (HLA) reference set was selected for the HTL epitopes prediction. Further, 15-mer length of the epitopes was retrieved and classified according to the percentile value of < 10.

### Prediction of linear B-cell epitopes

B-cell epitopes serve a crucial function in vaccine detection by inducing a humoral immune response that stimulates B cells for antibody formation. Using ABCpred servers, the antigens were exposed to linear B-cell epitope prediction^28^. With the overlapping filter turned on, a 16-mer window length was used for epitope identification using a recurrent neural network with a 0.51 threshold value. Only the most highly anticipated epitopes with scores greater than 0.5 were used to build the candidate vaccine.

### Prediction of the antigenicity of target protein

The need for identified vaccine candidates that contain antigenic properties is an important aspect of the vaccine detection process. Both VaxiJen v2.0 and ANTIGENpro servers were used to evaluate how antigenic the vaccination candidates were. ANTIGENpro, which determines the antigenicity of proteins using micro-array data. Based on cross-validation tests, the server’s accuracy with the combined dataset was determined to be 76%^29^. The epitopes are selected using antigenicity threshold value of more than 0.45, and positive IEDB immunogenicity values^30^. the antigenic evaluation of the chosen genes was carried out using the publicly available online VaxiJen 2.0 server^31^. The auto and cross-covariance (ACC) translation of protein sequences into uniform vectors of main amino acid characteristics is the foundation of the VaxiJen 2.0 server, which is used to assess the vaccine’s antigenicity. The VaxiJen program examines the physiochemical characteristics of proteins to determine if they are antigenic, mostly using the sequence alignment method^32^.

### Prediction of allergenicity and toxicity of target protein

Identification of allergens is essential to the vaccine’s detection. The allergenic characteristics of the proteins were evaluated by the AllerTOP v.2.0 server. By analyzing the physiochemical characteristics of proteins, the web server AllerTOP v2.0 develops machine learning algorithms for the categorization of allergens, including k nearest neighbors (kNN), auto- and cross-covariance (ACC) transformation, and amino acid E-descriptors. This method’s accuracy was reported to be 85.3% during fivefold cross-validation^33,34^. Protein sequences with non-allergic characteristics were chosen for additional examination. At last, the ToxinPred server was used to assess the toxicity of each epitope^35,36^ and non-toxic epitopes were selected. These features have also been confirmed for the vaccine’s overall construct.

### Virtual screening and molecular docking

The docking process is used to detect the promising ligands by calculating the binding affinity between the protein and ligands. We used Autodock Vina for the preparation, representation, visualization and analysis of the protein and ligand structures^37^. ZINC20 database is an Ultralarge scale database that contains more than 1.2 billion designed possible chemical complexes that are suitable for docking studies. The potential lead-like drugs database was downloaded in a MOL2 file from the ZINC20 database, then this file was developed and expanded to the main drugs’ groups using PowerShell in Windows 10^38^. These groups were used to create the database of the ligands for the docking process. Also, Autodock Vina was used to perform the docking analysis as it is easy to use, and it can represent the graphical reviews of the structures in an adjustable screen with many abilities to edit and clear the information of the interactions and positions of protein-ligand complexes. In addition, it shows the binding energy between protein-ligand complexes with every available pose and can prioritize them based on the lowest binding energy^17^.

### MD simulation

In the simulation process, every protein-ligand complex was centred in a cubic box with 10 Å distance to the surrounding edges (of boundary condition was defined in the x, y and z directions) and surrounded by TIP3P water molecules model. The salt concentration of the complex was maintained by adding 0.15 M of Na^+^ and Cl^−^ ions to the simulation box^39^, and particle-Ewald summation was used to evaluate the electrostatic interactions^40^, and for the estimation of VdW interactions a 10 Å cut-off was used. Then the resulting systems were energy-minimized for 50,000 steps through the steepest descent and conjugate gradient algorithms. After that, the systems were subjected to an NPT and NVT equilibrium phases for 100 ps for each process with integration step of 2fs^41,42^. Finally, the MD simulations production run for 100 ns with snapping every 10 ps for the analysis^43,44^. All production runs were performed on The Bibliotheca Alexandrina Supercomputing unit. GROMACS-2021.3 was used for the molecular dynamic simulation of the protein and protein-ligand complexes, with applying CHARMM27 force field in the estimation of the interactions^39,45,46^. GROMACS-2021.3 package was used for analysing the results of the MD simulations including the protein root mean square deviation (RMSD), root mean square fluctuation (RMSF), radius of gyration (RG), solvent accessible surface area (SASA), and hydrogen bonding (H-Bond)^47–49^.

### Binding free energy estimation

To accurately estimate the docking values, the binding affinity of ligands, the Molecular Mechanics Poisson–Boltzmann Surface Area (MM-PBSA) was used. MM-PBSA was used to accurately evaluate the binding free energies of the protein-ligand complexes. These data can be used to evaluate the molecular stability of the complexes. The binding free energies of the protein-ligand complexes can be estimated by:

∆G_binding_ = G_complex_ − G_protein_ − G_ligand_ ,

G_complex_ : the free energy of the protein–ligand complex.

G_protein_ : the free energy of protein.

G_ligand_ : the free energy of ligand.

The MM-PBSA results were used in the estimation of the binding of protein-ligand complexes and to estimate the potential interactions and binding sites^50–54^.

### Physiochemical and drug-likeness properties

The drug development process necessitates the evaluation of absorption, distribution, metabolism, and excretion (ADME) at progressively earlier stages of discovery, when the number of potential compounds is high but access to physical samples is constrained. Swiss-ADME server was used in the estimation of the physicochemical properties and the drug-likeness properties of the selected ligands^55^. Swiss-ADME server could calculate the molecular weight, H-bond acceptors, H-bod donors, and Lipinski’s rule of five values which are crucial in detecting the most suitable drug. It includes proprietary methods such as the BOILED-Egg, iLOGP, and Bioavailability Radar. ProTox III and ADMET-AI servers were used to discover the drug like properties of the ligands^56,57^.

## Data Availability

All the data used in the study were included in the article.

## References

[1] Huang, C. et al. Clinical features of patients infected with 2019 novel coronavirus in Wuhan, China. *Lancet* **395**, 497–506 (2020). 31986264 10.1016/S0140-6736(20)30183-5 PMC7159299

[2] Li, Q. et al. Early transmission dynamics in Wuhan, China, of novel coronavirus–infected pneumonia. *N Engl. J. Med.* **382**, 1199–1207 (2020). 31995857 10.1056/NEJMoa2001316 PMC7121484

[3] Guo, Y. R. et al. The origin, transmission and clinical therapies on coronavirus disease 2019 (COVID-19) outbreak - an update on the status. *Mil Med. Res.* **7** (1), 11 (2020). 32169119 10.1186/s40779-020-00240-0 PMC7068984

[4] Zhou, P. et al. A pneumonia outbreak associated with a new coronavirus of probable Bat origin. *Nature* **579**, 270–273 (2020). 32015507 10.1038/s41586-020-2012-7 PMC7095418

[5] Lu, R. et al. Genomic characterization and epidemiology of 2019 novel coronavirus: implications for virus origins and receptor binding. *Lancet* **S0140-6736** (20), 30251–30258 (2020). 10.1016/S0140-6736(20)30251-8 PMC7159086 32007145

[6] Abdelkader, A. et al. In-Silico targeting of SARS-CoV-2 NSP6 for drug and natural products repurposing. *Virology* **573**, 96–110 (2022). 35738174 10.1016/j.virol.2022.06.008 PMC9212324

[7] Lubin, J. H. et al. Evolution of the SARS-CoV-2 proteome in three dimensions (3D) during the first six months of the COVID-19 pandemic. (2020). 10.1002/prot.26250 PMC8661935 34580920

[8] Pandey, P., Prasad, K., Prakash, A. & Kumar, V. Insights into the biased activity of dextromethorphan and haloperidol toward SARS-CoV-2 NSP6: in Silico binding mechanistic analysis. *J. Mol. Med. (Berl)*. **98**, 1659–1673 (2020). 32965508 10.1007/s00109-020-01980-1 PMC7509052

[9] Morais, I. J. et al. The global population of SARS-CoV-2 is composed of six major subtypes. *Sci. Rep.* **10**, 18289 (2020). 33106569 10.1038/s41598-020-74050-8 PMC7588421

[10] Cottam, E. M., Whelband, M. C. & Wileman, T. Coronavirus NSP6 restricts autophagosome expansion. *Autophagy* **10**, 1426–1441 (2014). 24991833 10.4161/auto.29309 PMC4203519

[11] van der Hoeven, B. et al. Biogenesis and architecture of arterivirus replication organelles. *Virus Res.* **220**, 70–90 (2016). 27071852 10.1016/j.virusres.2016.04.001 PMC7111217

[12] Zhang, J., Lan, Y. & Sanyal, S. Membrane heist: coronavirus host membrane remodeling during replication. *Biochimie* **179**, 229–236 (2020). 33115667 10.1016/j.biochi.2020.10.010 PMC7585727

[13] Santerre, M., Arjona, S. P., Allen, C. N., Shcherbik, N. & Sawaya, B. E. Why do SARSCoV-2 NSPs rush to the ER? *J. Neurol.* 1–10 (2020). 10.1007/s00415-020-10197-8 PMC7461160 32870373

[14] De Gordon, Jang, G. M. et al. A SARS-CoV-2 protein interaction map reveals targets for drug repurposing. *Nature* **583**, 459–468 (2020). 32353859 10.1038/s41586-020-2286-9 PMC7431030

[15] Edache, E. I., Uzairu, A., Mamza, P. A. & Shallangwa, G. A. QSAR, homology modeling, and Docking simulation on SARS-CoV-2 and Pseudomonas aeruginosa inhibitors, ADMET, and molecular dynamic simulations to find a possible oral lead candidate. *J. Genet. Eng. Biotechnol.* **20**, 88 (2022). 35730025 10.1186/s43141-022-00362-z PMC9205150

[16] Edache, E. I., Uzairu, A. & Mamza, P. A. Shallangwa theoretical investigation of the Cooperation of Iminoguanidine with the enzymes-binding domain of covid-19 and bacterial lysozyme inhibitors and their Pharmacokinetic properties. *J. Mex. Chem. Soc.* **66** (4), 513–542 (2022).

[17] Clark, A. M. & Labute, P. 2D depiction of protein–ligand complexes. *J. Chem. Inf. Model.* **47**, 1933–1944 (2007). 17715911 10.1021/ci7001473

[18] Prakash A, Kumar V, Meena NK, et al. Elucidat ion of the structural stability and dynamics of heterogeneous intermediateensembles in unfolding pathway of the N-terminal domain of TDP-43. *RSC Adv.* **8** (35), 19835–45 (2018). 10.1039/c8ra03368d PMC9088055 35548664

[19] McGuffin, L. J., Bryson, K. & Jones, D. T. The PSIPRED protein structure prediction server. *Bioinformatics* **16** (4), 404–405 (2000). 10869041 10.1093/bioinformatics/16.4.404

[20] Heo, L. & Feig, M. *Modeling of Severe Acute Respiratory Syndrome Coronavirus 2 (SARS-CoV-2) Proteins by Machine Learning and Physics-Based Refiement* (2020).

[21] Senior, A. W. et al. Improved protein structure prediction using potentials from deep learning. *Nature* **577**, 706–710 (2020). 31942072 10.1038/s41586-019-1923-7

[22] Jumper, J. et al. Highly accurate protein structure prediction with alphafold. *Nature* **596**, 583–589 (2021). 34265844 10.1038/s41586-021-03819-2 PMC8371605

[23] Shuvo, M. H. & Gulfam, M. Debswapna Bhattacharya, DeepRefiner: high-accuracy protein structure refinement by deep network calibration. *Nucleic Acids Res.* **49** (W1), W147–W152. (2021). 10.1093/nar/gkab361 PMC8262753 33999209

[24] Colovos, C. YeatesTO. Verification of protein structures: patterns of nonbonded atomic interactions. (1993). 10.1002/pro.5560020916 PMC2142462 8401235

[25] Larsen, M. V. et al. Large-scale validation of methods for cytotoxic T-lymphocyte epitope prediction. *BMC Bioinform*. **8**, 424 (2007). 10.1186/1471-2105-8-424 PMC2194739 17973982

[26] Peters, B., Bulik, S., Tampe, R., Van Endert, P. M. & Holzhütter, H. G. Identifying MHC class I epitopes by predicting the TAP transport efciency of epitope precursors. *J. Immunol.* **171**, 1741–1749 (2003). 12902473 10.4049/jimmunol.171.4.1741

[27] Zhang, Q. et al. Immune epitope database analysis resource (IEDB-AR). *Nucleic Acids Res.* **36**, W513–W518 (2008). 18515843 10.1093/nar/gkn254 PMC2447801

[28] Saha, S. & Raghava, G. P. S. Prediction of continuous B-cell epitopes in an antigen using recurrent neural network. *Prot. Struct. Funct. Bioinform*. **65**, 40–48 (2006). 10.1002/prot.21078 16894596

[29] Magnan, C. N. et al. High-throughput prediction of protein antigenicity using protein microarray data. *Bioinformatics* **26**, 2936–2943 (2010). 20934990 10.1093/bioinformatics/btq551 PMC2982151

[30] Khan, M. T. et al. Immunoinformatics and molecular modeling approach to design universal multi-epitope vaccine for SARS-CoV-2. *Inf. Med. Unlocked*. **24**, 100578 (2021). 10.1016/j.imu.2021.100578 PMC8057924 33898733

[31] Doytchinova, I. A., Flower, D. R. & VaxiJen A server for prediction of protective antigens, tumour antigens and subunit vaccines. *BMC Bioinform*. **8**, 4 (2007). 10.1186/1471-2105-8-4 PMC1780059 17207271

[32] Pandey, R. K., Ojha, R., Aathmanathan, V. S., Krishnan, M. & Prajapati, V. K. Immunoinformatics approaches to design a novel multi-epitope subunit vaccine against HIV infection. *Vaccine* **36**, 2262–2272 (2018). 29571972 10.1016/j.vaccine.2018.03.042

[33] Dimitrov, I., Bangov, I., Flower, D. R. & Doytchinova, I. AllerTOP v.2—a server for in Silico prediction of allergens. *J. Mol. Model.* **20**, 2278 (2014). 24878803 10.1007/s00894-014-2278-5

[34] Dimitrov, I., Naneva, L., Doytchinova, I. & Bangov, I. AllergenFP: allergenicity prediction by descriptor fngerprints. *Bioinformatics* **30**, 846–851 (2014). 24167156 10.1093/bioinformatics/btt619

[35] Shahid, F., Ashfaq, U. A., Javaid, A. & Khalid, H. Immunoinformatics guided rational design of a next generation multi epitope based peptide (MEBP) vaccine by exploring Zika virus proteome. *Infect. Genet. Evol.* **80**, 104199 (2020). 31962160 10.1016/j.meegid.2020.104199

[36] Gupta, S. et al. In Silico approach for predicting toxicity of peptides and proteins. *PLoS One*. **8**, e73957 (2013). 24058508 10.1371/journal.pone.0073957 PMC3772798

[37] Mount, D. W. Using the basic local alignment search tool (BLAST). *Cold Spring Harb. Protoc.* **2007**, pdb.top17. (2007). 10.1101/pdb.top17 21357135

[38] Irwin, J. J. et al. ZINC20-A free Ultralarge-Scale chemical database for ligand discovery. *J. Chem. Inf. Model.* **60** (12), 6065–6073 (2020). 33118813 10.1021/acs.jcim.0c00675 PMC8284596

[39] Huang, J. et al. CHARMM36m: an improved force field for folded and intrinsically disordered proteins. *Nat. Methods*. **14**, 71–73 (2017). 27819658 10.1038/nmeth.4067 PMC5199616

[40] Darden, T., York, D. & Pedersen, L. Particle mesh ewald: an N·log(N) method for Ewald sums in large systems. *J. Chem. Phys.* **98**, 10089–10092 (1993).

[41] Bussi, G., Donadio, D. & Parrinello, M. Canonical sampling through velocity rescaling. *J. Chem. Phys.* **126**, 014101 (2007). 17212484 10.1063/1.2408420

[42] Parrinello, M. & Rahman, A. Crystal structure and pair potentials: a molecular-dynamics study. *Phys. Rev. Lett.* **45**, 1196–1199 (1980).

[43] Singh, R. et al. Delineating the conformational dynamics of intermediate structures on the unfolding pathway of beta-lactoglobulin in aqueous urea and dimethyl sulfoxide. *J. Biomol. Struct. Dyn.* 1–10. (2019). 10.1080/07391102.2019.1695669 31744390

[44] Prakash, A. et al. Elucidation of stable intermediates in urea-induced unfolding pathway of human carbonic anhydrase IX. *J. Biomol. Struct. Dyn.* **36** (9), 2391–2406 (2018). 28705076 10.1080/07391102.2017.1355847

[45] Kutzner, C. et al. More Bang for your buck: improved use of GPU nodes for GROMACS 2018. *J. Comput. Chem.* **40**, 2418–2431 (2019). 31260119 10.1002/jcc.26011

[46] Vanommeslaeghe, K., Raman, E. P. & MacKerell, A. D. Jr. Automation of the CHARMM general force field (CGenFF) II: assignment of bonded parameters and partial atomic charges. *J. Chem. Inf. Model.* **52**, 3155–3168 (2012). 23145473 10.1021/ci3003649 PMC3528813

[47] Laberge, M. & Yonetani, T. Molecular dynamics simulations of hemoglobin A in different States and bound to DPG: effector-linked perturbation of tertiary conformations and HbA concerted dynamics. *Biophys. J.* **94**, 2737–2751 (2008). 18096633 10.1529/biophysj.107.114942 PMC2267116

[48] Prakash, A. et al. Structural heterogeneity in RNA recognition motif 2 (RRM2) of TAR DNA-binding protein 43 (TDP-43): clue to amyotrophic lateral sclerosis. *J. Biomol. Struct. Dyn.* 1-11. (2020). 10.1080/07391102.2020.1714481 31914861

[49] Prakash, A. & Luthra, P. M. Insilico study of the A(2A)R-D (2)R kinetics and interfacial contact surface for heteromerization. *Amino Acids*. **43**, 1451–1464 (2012). 22278740 10.1007/s00726-012-1218-x

[50] Wang, E. et al. End-point binding free energy calculation with MM/PBSA and MM/GBSA: strategies and applications in drug design. *Chem. Rev.* **119**, 9478–9508 (2019). 31244000 10.1021/acs.chemrev.9b00055

[51] Batt, S. M. et al. Structural basis of Inhibition of Mycobacterium tuberculosis DprE1 by Benzothiazinone inhibitors. *Proc. Natl. Acad. Sci. U S A*. **109**, 11354–11359 (2012). 22733761 10.1073/pnas.1205735109 PMC3396498

[52] Sastry, G. M. et al. Protein and ligand preparation: parameters, protocols, and influence on virtual screening enrichments. *J. Comput. Aided Mol. Des.* **27**, 221–234 (2013). 23579614 10.1007/s10822-013-9644-8

[53] Wang, C. et al. Calculating protein-ligand binding affinities with MMPBSA: method and error analysis. *J. Comput. Chem.* **37**, 2436–2446 (2016). 27510546 10.1002/jcc.24467 PMC5018451

[54] Ravikumar, Y. et al. Silico molecular docking and dynamics simulation analysis of potential histone lysine methyl transferase inhibitors for managing β -thalassemia. *Molecules* 287266. (2023). 10.3390/molecules28217266 PMC10650625 37959685

[55] Banerjee, P., Kemmler, E., Dunkel, M. & Preissner, R. ProTox 3.0: a webserver for the prediction of toxicity of chemicals. *Nucleic Acids Res.* **52** (W1), W513–W520 (2024). 38647086 10.1093/nar/gkae303 PMC11223834

[56] Swanson, K. et al. James Zou, ADMET-AI: a machine learning ADMET platform for evaluation of large-scale chemical libraries. *Bioinformatics* **40** (7), btae416 (2024). 10.1093/bioinformatics/btae416 PMC11226862 38913862

[57] Roy, H., Nayak, B. S. & Nandi, S. *Silico* factorial screening and optimization of Chitosan based gel for urapidil loaded microparticle using reduced factorial design. *Comb. Chem. High. Throughput Screen.* **23** (10), 1049–1063 (2020). 32598248 10.2174/1386207323666200628110552

